# Endothelial TRIM35‐Regulated MMP10 Release Exacerbates Calcification of Vascular Grafts

**DOI:** 10.1002/advs.202409641

**Published:** 2025-01-27

**Authors:** Yiming Leng, Wei Wang, Jun Lu, Jingyuan Chen, Xuliang Chen, Yalan Li, Jie Wang, Yuanyuan Liu, Qian Tan, Wenjing Yang, Youxiang Jiang, Peiyuan Huang, Jingjing Cai, Hong Yuan, Liang Weng, Qingbo Xu, Yao Lu

**Affiliations:** ^1^ Clinical Research Center Postdoctoral Station of Clinical Medicine The Third Xiangya Hospital Central South University Changsha 410013 P. R. China; ^2^ Department of Laboratory Medicine The Third Xiangya Hospital Central South University Changsha 410013 P. R. China; ^3^ Department of Cardiovascular Surgery Xiangya Hospital Central South University Changsha 410028 P. R. China; ^4^ MRC Integrative Epidemiology Unit (IEU) Bristol Medical School University of Bristol Oakfield House, Oakfield Grove Bristol BS8 2BN UK; ^5^ Department of Pathology School of Basic Medical Sciences Peking University Third Hospital Peking University Health Science Center Beijing 100083 P. R. China; ^6^ Department of Cardiology, the First Affiliated Hospital School of Medicine Zhejiang University Hangzhou 310058 P. R. China; ^7^ Life Sciences & Medicine King's College London London SE1 8WA UK

**Keywords:** calcification, endothelial cells, MMP10, smooth muscle cells, TRIM35

## Abstract

Vascular calcification is a highly regulated process in cardiovascular disease (CVD) and is strongly correlated with morbidity and mortality, especially in the adverse stage of vascular remodeling after coronary artery bypass graft surgery (CABG). However, the pathogenesis of vascular graft calcification, particularly the role of endothelial‐smooth muscle cell interaction, is still unclear. To test how ECs interact with SMCs in artery grafts, single‐cell analysis of wild‐type mice is first performed using an arterial isograft mouse model and found robust cytokine‐mediated signaling pathway activation and SMC proliferation, together with upregulated endothelial tripartite motif 35 (TRIM35) expression. Unexpectedly, severe SMC calcification in artery grafts is found in TRIM35 conditional endothelial knockout (cKO) mice. Calcified medium (comprising calcium chloride and beta‐glycerophosphate)‐induced calcium deposition in vitro is also found in SMCs cocultured with TRIM35 knockout endothelium. This extraordinary phenomenon is further confirmed to be induced by increased MMP10 secretion. Mechanistically, endothelial TRIM35 inhibits MMP10 expression and secretion by promoting K63‐linked ubiquitination of RelB and maintaining its nuclear localization, consequently inhibiting nuclear transcription of MMP10 through the noncanonical NF‐κB signaling pathway. Targeting MMP10 in situ in arterial isografts can effectively alleviate vascular calcification caused by conditional endothelial TRIM35 knockout. These findings demonstrated that TRIM35 inhibited vascular calcification during arterial isograft remodeling, a process that is driven by the aberrant secretion of endothelial MMP10. Targeting MMP10 pathway may be a potential therapeutic strategy for vascular calcification in vessel grafts.

## Graphical abstract

Arterial bypass graft is routinely used for the patients with coronary artery infarction, but is limited by progressive vascular restenosis and occlusion occurs, in which vascular calcification is a key issue. Endothelial TRIM35 can protect the isograft vascular from calcification by activating RelB ubiquitination that inhibits MMP10 secretion. Targeting endothelial MMP10 function is a potential therapy against graft failure.

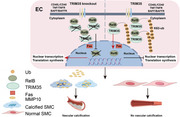

## 1 Introduction

Coronary artery bypass graft surgery (CABG) has become a currently life‐saving therapy for patients with coronary atherosclerosis disease.^[^ ^1^, ^2^, ^3^ ^]^ However vascular graft remodeling and its resultingly lumen occlusion are the leading cause of secondary myocardial ischemia and graft failure, which brings main challenge to haemodynamic reconstruction and severely affects the prognosis of CABG patients.^[^ ^4^ ^]^ Evidence discloses that endothelium activation and dysfunction initiate graft remodeling by inducing SMC abnormal proliferation and accumulation in neointima.^[^ ^5^, ^6^, ^7^ ^]^ Nevertheless, vascular calcification also shows its prevalence in CABG and other cardiovascular diseases (CVD) with highly correlation of CVD mortality,^[^ ^8^, ^9^ ^]^ which is a complicated and active remodeling process involving the deposition of hydroxyapatite with a high degree of crystallization.^[^ ^10^ ^]^ Vascular calcification promotes a loss of arterial compliance and an increase in pulse pressure and serves as a marker of atherosclerotic plaque severity.^[^ ^11^, ^12^ ^]^ To date, several mechanisms have been explored to explain calcification formation^[^ ^13^, ^14^ ^]^ but the initiating and core mechanisms of vascular calcification in various diseases are still diverse: for instance the calcification process in atherosclerosis is completely different from medial calcification caused by primary damage to elastic fibers.^[^ ^10^ ^]^ However, considering the variability of pathogenic factors,^[^ ^15^ ^]^ effective pharmacological therapies and targets for reversing or preventing calcification are lacking.

The core cell type of vascular calcification is SMC, which plays a vital role in maintaining blood pressure and regulating the extracellular matrix (ECM) of blood vessels.^[^ ^16^ ^]^ Under the resting state, SMC shows a slow‐proliferated, functionally contractile phenotype. Due to its phenotype plasticity, it could turn to decrease contractile function and increase proliferation and ECM regulation to adjust injury and present synthetic and osteochondrogenic phenotype.^[^ ^17^ ^]^ During this period, which often induced by oxidative stress and inflammation in atherosclerosis, EC‐SMC interaction and crosstalk show great effect on vascular calcification. Enhancing neoangiogenesis is necessary for areas of calcium deposition, newly formed micro vessels proliferated and surrounded calcified deposits of which degree highly correlated with the severity of the lesion.^[^ ^18^ ^]^ Advanced calcification may also induce endothelium hyperplasia which in turn aggravate vascular remodeling.^[^ ^19^ ^]^ Although the precise regulation mechanism is still controversial, some unique genetic findings have been identified, such as blood vessels normally express pyrophosphate and matrix protein which mainly by ECs to tame calcification.^[^ ^20^, ^21^ ^]^ In general, challenges still remain to comprehend the intricate mechanisms on how ECs directly regulate SMC calcification in vascular grafts.

Endothelial cells play a crucial role in vascular graft remodeling,^[^ ^22^, ^23^ ^]^ in which ubiquitination process is a key event.^[^ ^24^, ^25^, ^26^ ^]^ Previous studies have been shown that tripartite motif 35 (TRIM35)^[^ ^27^, ^28^ ^]^ can ubiquitinate and degrade nuclear pyruvate kinase isoform M2 (PKM2) in cardiomyocytes, causing GATA4/6 destabilization and p53 activation in dilated human heart failure.^[^ ^29^ ^]^ TRIM35 contains an N‐terminal Really Interesting New Gene (RING) domain, one or two B‐boxes (B1/B2), and a coiled coil (CC) domain (RBCC).^[^ ^30^ ^]^ The main function of TRIM35 occurs through its RING domain, which allows TRIM35 to act as a ubiquitin E3 ligase and recognize specific substrates during ubiquitination.^[^ ^31^ ^]^ Although increasing evidences indicate that TRIM proteins are highly associated with various cardiac processes and pathologies though E3 ligase function and ubiquitination,^[^ ^32^, ^33^, ^34^ ^]^ it still remains unknown whether TRIM35 expressed in endothelial cells regulates SMC calcification and vascular remodeling in artery grafts. In the present study, we aim to investigate the role of endothelial TRIM35 in vascular calcification after artery isograft surgery, finding that TRIM35 knockout in ECs resulted in severe SMC calcification induced by the paracrine effect of endothelial MMP10 (matrix metalloproteinase 10) release. Mechanistically endothelial TRIM35 promoted the K63‐linked ubiquitination of RelB and the maintenance of its nuclear localization, causing the noncanonical NF‐κB signaling pathway activation which led to inhibition of MMP10 transcription and expression. Additionally, we illustrated MMP10 as a strong predictor for unfavorable prognosis in CABG patients. Targeting MMP10 can effectively ameliorate vascular calcification in artery isografts. Our findings highlight the critical role of endothelial TRIM35‐derived MMP10, which may provide novel insights into understanding of vascular calcification after CABG.

## 2 Methods

### 2.1 Availability of Data

All the scRNA‐seq data are available in the Gene Expression Omnibus (GSE140812, GSE140968, and GSE149452).

This Manuscript was prepared using Framingham Heart Study (FHS) offspring Research Materials obtained from the NHLBI Biologic Specimen and Data Repository Information Coordinating Center and does not necessarily reflect the opinions or views of the FHS or the NHLBI. The data are available from BioLINCC (https://biolincc.nhlbi.nih.gov/home/).

An expanded Materials and Methods section can be found in the Supplemental Material.

## 3 Results

### 3.1 Endothelial TRIM35 is Upregulated after CABG

**Figure 1:**
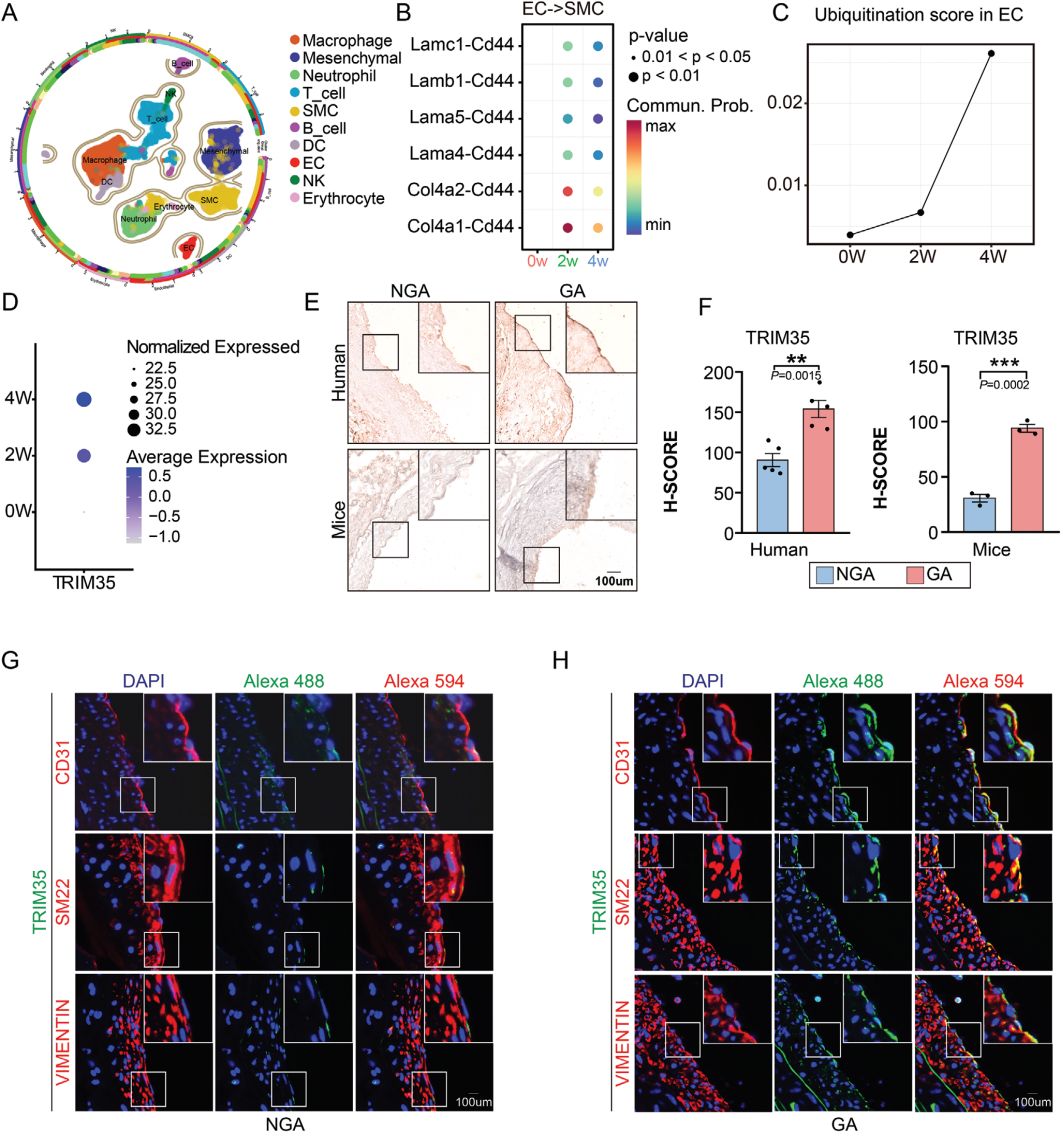
Endothelial TRIM35 is upregulated after CABG. A) UMAP plot for 10 major cell clusters in the aortic graft, *n* = 39,787 cells. B) Dotplot for ligand‐receptor interaction pairs between EC ligand‐expressing and SMC receptor‐expressing cluster. C) Line chart showing changes of ubiquitination score in EC cluster after surgery. D) Dotplot displaying changes of TRIM35 expression level in EC cluster after surgery. E,F) Immunohistochemistry (IHC) staining for TRIM35 in arteries from patients with graft atherosclerosis (GA) post‐CABG and non‐graft atherosclerosis (NGA) post‐CABG, as well as on arteries from mice with GA and NGA. (F) H‐Score for TRIM35 in human and mice intima, *n* = 5 in human group and *n* = 3 in mice group. G,H) Immunofluorescence (IF) staining for TRIM35 (green) and CD31 (red), SM22 (red) or VIMENTIN (red) in arteries from mice with graft atherosclerosis (GA) post‐CABG and non‐graft atherosclerosis (NGA) post‐CABG. Yellow indicates the co‐localization region. Data are means and SEM; *p < 0.05, **p < 0.01, ***p < 0.001.

In previous research endeavors to investigate vascular remodeling, we established murine vascular allograft and isograft models.^[^ ^6^, ^22^ ^]^ To validate the changes of EC‐SMC interaction in the transplanted vascular tissue throughout the remodeling process, we first analyzed single‐cell RNA sequencing (scRNA‐seq) data of vascular isografts (**Figure** **1**A–D; Figures S1 and S2, Supporting Information). Our analysis involved scRNA‐seq data from three distinct groups of C57BL/6J mice: a sham surgery group, a 2‐week transplant group, and a 4‐week transplant group (GSE140812; GSE140968; GSE149452).^[^ ^6^, ^22^ ^]^ We determined the proportions of various cell types within each group through clustering analysis and annotation (Figure 1A; Figure S1, Supporting Information). Cell cluster analysis yielded noteworthy findings including a significant reduction after vascular transplantation (0w‐2w) in mesenchymal cells, vascular smooth muscle cells (VSMCs), and endothelial cells (ECs), implying injury under mechanical and inflammatory response, which were gradually increased over restore time (Figure S2, Supporting Information). In addition, it was coupled with an increase in immunoregulatory cells (e.g., macrophages, B cells, and T cells) in the transplant groups (Figure S2A, Supporting Information). Gene Ontology (GO) enrichment analysis and Gene set enrichment analysis (GSEA) indicated the significant activation of pathways related to vascular generation and a marked decrease in the activation of extracellular matrix‐related pathways after transplantation (Figure S3A‐C, Supporting Information). Ligand‐receptor analysis highlighted extensive interactions between ECs and VSMCs after transplantation (Figure 1B), which exhibited EC response to injured VSMCs in thickened intima to promote VSMCs proliferation and migration.^[^ ^35^, ^36^ ^]^ These suggested EC‐mediated VSMC phenotypic switching and restoration may also play a significant role in vascular remodeling. Further subgroup analysis similarly revealed phenotypic transformation in the transplanted VSMCs (Figure S3D,E, Supporting Information).

Furthermore, to uncover the regulatory function of TRIM35 in EC‐SMC interaction during vascular remodeling, we examined ubiquitination‐related genes in the EC cluster, for ubiquitination plays a crucial role in endothelial dysfunction and is involved in the progression of a diverse diseases caused by endothelial impairment.^[^ ^37^ ^]^ Interestingly, we found higher ubiquitination score of ECs after transplantation (Figure 1C). Moreover, TRIM35 which has been lately confirmed to be involved in heart failure through myocardial cells^[^ ^29^ ^]^ exhibited a trend toward increased expression within the transplanted EC cluster (Figure 1D). To verify this phenomenon we collected atherosclerotic arteries from patients after CABG, as well as arteries from mouse isograft models. TRIM35 expression was indeed obviously elevated in both patients and mice arteries during atherosclerosis and artery remodeling (Figure 1E,F; Figure S4, Supporting Information). In parallel, immunofluorescence staining confirmed that increased TRIM35 expression occurred primarily in the ECs of diseased vessels indeed (Figure 1G,H). These findings further suggested a potential involvement of the TRIM35 in the vascular remodeling process, although its precise role, whether promotional or protective, remains uncertain. Taken together, we found that TRIM35 may play a pivotal role in vascular remodeling which EC‐mediated VSMC phenotypic switching receive highly relevance, but the underlying mechanisms need to be further explored.

### 3.2 Endothelial TRIM35 Knockout Exacerbates Vascular Calcification

**Figure 2:**
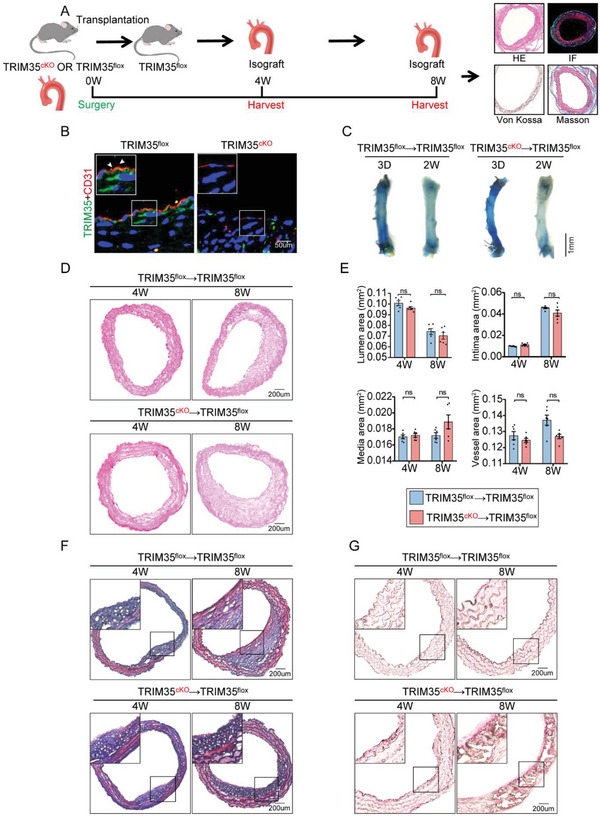
Endothelial TRIM35 Knockout Exacerbates Vascular Calcification. A) Schematic procedure for transplanting TRIM35flox or TRIM35cKO aortic segments to TRIM35flox mice for 4 or 8 weeks. B) Immunofluorescence (IF) staining for TRIM35 (green) and CD31 (red) in arteries from TRIM35flox or TRIM35cKO mice. C) Evan’ s Blue staining in arterial grafts for 3 days or 2 weeks, *n* = 3. D,E) Hematoxylin and Eosin (HE) staining of isograft arteries from 4W or 8W TRIM35flox and TRIM35cKO mice (D). The above images were all taken under a 10X objective lens. Quantification of neointimal and luminal areas were shown in E, *n* = 6 in each group. Data are means and SEM. F) Masson staining of isograft arteries from 4W or 8W TRIM35flox and TRIM35cKO mice. The above images were all taken under a 10X objective lens. G) Von Kossa and Nuclear Fast Red staining of isograft arteries from 4W or 8W TRIM35flox and TRIM35cKO mice. The above images were all taken under a 20X objective lens. Data are means and SEM; *p < 0.05, **p < 0.01, ***p < 0.001.

To further investigate the role of endothelial TRIM35 in posttransplant vascular remodeling, specifically to determine whether TRIM35 exerts a protective effect in this context, we generated conditional endothelial TRIM35 knockout mice (hereafter referred to as TRIM35cKO mice) through a breeding strategy involving TRIM35flox mice and Cdh5‐iCre mice (Figure S5, Supporting Information). Subsequently, we transplanted both TRIM35cKO and TRIM35flox mice arteries into TRIM35flox recipient mice (**Figure** **2**A,B). Approximate vascular permeability was observed no matter if TRIM35 was deleted in ECs, as indicated by Evan's blue and en face staining (Figure 2C). However, compared to those in the TRIM35flox group, the expression of ICAM1 and VCAM1 and immune cell infiltration were significantly greater in the TRIM35cKO group indicating vascular inflammation. eNOS was observed downregulated in TRIM35cKO group suggesting poorer arterial function during endothelial repair which symbolized by CD144 upregulation (Figure S6, Supporting Information). HE staining revealed that, compared with TRIM35flox mice, isografts from TRIM35cKO mice did not exhibit additional exacerbation of vascular stenosis after transplantation; there was no significant difference in lumen area, intima area, media area, and vessel area (Figure 2D,E). We also performed diversified histological validation and found a trend toward an increase in collagen fiber content within the thickened intima (Figure 2F). To further explain this anomalous phenomenon, we using Von Kossa staining exhibited remarkably VSMCs calcification in TRIM35cko grafts at the 8‐week mark compared with TRIM35flox, whereas no signs of calcification were evident at 4 weeks (Figure 2G). Taken together, our results emphasized the involvement of endothelial TRIM35 in posttransplant vascular remodeling. Endothelial TRIM35 knockout markedly induced vascular calcification causing isogaft vascular stenosis, which had never been reported before.

### 3.3 TRIM35 Knockout Induces EC‐Driven VSMC Calcification During Phenotype Switching

**Figure 3:**
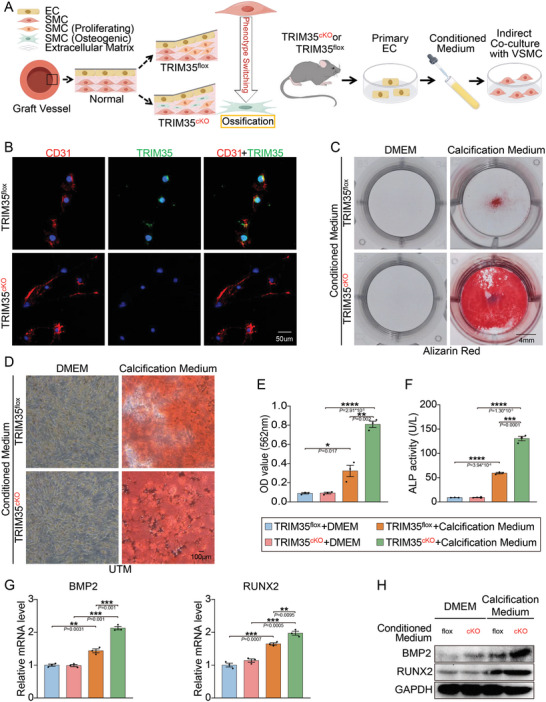
TRIM35 Knockout Induces EC‐driven VSMC Calcification During Phenotype Switching. A) Schematic model for strategy to explore the effect of EC on VSMC osteogenic switching. B) IF staining for TRIM35 (green) and CD31 (red) of pEC isolated from TRIM35flox and TRIM35cKO mice. C–E) Alizarin Red staining for calcium nodules in pVSMC indirectly co‐cultured with TRIM35flox or TRIM35cKO pEC (C). Perspective under the microscope (UTM) was shown in D. Quantification of Alizarin Red staining in the pVSMCs was shown in E, *n* = 3 in each group. F) Quantification of alkaline phosphatase (ALP) activity of pVSMC co‐cultured with conditioned medium extracted from TRIM35flox or TRIM35cKO pEC and DMEM or calcification medium, *n* = 3 in each group. G,H) QPCR (G) and Western‐blotting (H) illustrating the RNA and protein levels of BMP2 and RUNX2 in pVSMCs co‐cultured with conditioned medium extracted from TRIM35flox or TRIM35cKO pECs and treated with either DMEM or calcification medium. Each group consisted of *n* = 3 samples. Data are means and SEM, *p < 0.05, **p < 0.01, ***p < 0.001, ****p < 0.0001.

Given the extraordinary view of TRIM35 ablation derived calcification in isograft, we then speculated that endothelial TRIM35 may prevent VSMC from calcification. To test this hypothesis, we isolated primary endothelial cells (pECs) and primary vascular smooth muscle cells (pVSMCs) to explore effect of TRIM35 in EC‐VSMC interaction during vascular calcification. Using indirect coculture of pECs and pVSMCs with conditioned medium generated from pECs and exposing to calcium deposition‐induced situation (3 mM calcium chloride and 10 mM beta‐glycerophosphate in DMEM) (**Figure** **3**A,B). Assessment of calcified nodules via Alizarin Red staining (Figure 3C–E) and the alkaline phosphatase (ALP) assay (Figure 3F) both revealed that conditioned medium from TRIM35cKO pECs significantly promoted osteogenic calcium deposition. And the expression of osteogenic marker proteins (BMP2 and RUNX2) in pVSMCs after calcified stimulation are all upregulated (Figure 3G,H). These observations further supported that endothelial TRIM35 knockout induced VSMC calcification in vitro.

The calcification of VSMCs represents a specific type of VSMC phenotypic transition.^[^ ^12^, ^38^ ^]^ We sequentially assessed markers related to VSMC phenotypic transition in TRIM35cKO transplanted arteries, showing that significantly elevated synthetic and osteogenic phenotype numbers of among the increasing VSMCs (Figure S8, Supporting Information). In addition, we also found increased calcification markers (BMP2 and RUNX2) in the transplanted arteries (Figure S9D,E, Supporting Information). These robust findings revealed that TRIM35 deletion in the endothelium triggered VSMC phenotypic switching and calcification through EC‐SMC communication, resulting in calcium deposition.

### 3.4 MMP10 Upregulation in TRIM35‐KO ECs Stimulated VSMC Calcification

**Figure 4:**
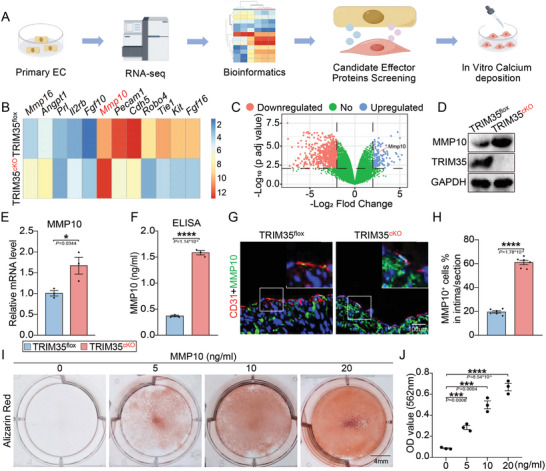
MMP10 Upregulation in TRIM35‐KO ECs Stimulated VSMC Calcification. A) Schematic model illustrating strategies for screening and validating potential effector proteins involved in EC‐mediated VSMC osteogenic switching. B) Heatmap displaying the top six cytokine related genes with the highest levels of upregulation and downregulation between TRIM35flox and TRIM35cKO pEC. C) Volcano plot showing gene features TRIM35flox and TRIM35cKO pEC. Selected DEGs (with P adj value < 0.05; log10 (fold change) >2 or log2 (fold change) ←2) were highlighted in blue or red. D,E) Western‐blotting (D) and qPCR (E) illustrating MMP10 protein and RNA level of TRIM35flox and TRIM35cKO pEC, *n* = 3 in each group. F) Enzyme‐linked immunosorbent assay (ELISA) was performed to measure secretion level of MMP10 in conditioned medium collected from TRIM35flox and TRIM35cKO pEC. G,H) IF staining (G) for MMP10 (green) and CD31 (red) in isografts from TRIM35flox or TRIM35cKO mice. Quantification of positive cell counting in intima layer of each section (H), *n* = 6 in each rgroup. I,J) MMP10 in vitro stimulation experiment. Alizarin Red staining for calcium nodules in pVSMC stimulated by MMP10 was shown in I. Quantification of Alizarin Red staining in the pVSMCs was shown in J, *n* = 3. Data are means and SEM, *p < 0.05, **p < 0.01, ***p < 0.001, ****p < 0.0001.

Next, to explore the mechanism of VSMC calcification driven by endothelial TRIM35 knockout, we performed RNA sequencing (RNA‐seq) on TRIM35cKO and TRIM35flox pECs and conducted a thorough analysis. Herein, we present the cytokine‐related genes exhibiting the highest and lowest differential expression between TRIM35flox and TRIM35cKO pECs (**Figure** **4**A). There was a significant reduction in the expression of endothelial cell‐related markers, including Cdh5, Pecam1, Tie1, and Kit in TRIM35cKO pECs. Notably, we found MMP10 was remarkable upregulated (Figure 4B,C). As we know osteogenic transformation of VSMCs often accompanies remodeling of the ECM,^[^ ^39^, ^40^ ^]^ and the MMP protein family plays a pivotal role in the degradation of ECM components, thereby facilitating processes such as cell proliferation, differentiation, migration and tissue regeneration.^[^ ^41^, ^42^ ^]^ Although the MMP protein family has previously been shown to be associated with vascular calcification,^[^ ^43^ ^]^ the specific mechanism of MMP10 in this process has not been explored. Thus, we focused on the role of MMP10 and hypothesized it might be released through endothelial paracrine signaling and induced VSMC calcification. Additionally, we measured the mRNA and secretion of MMP10 in TRIM35flox and TRIM35cKO pECs, observing remarkable increased upregulaton of MMP10 after endothelial TRIM35 knockout (Figure 4D–F). Immunofluorescence staining was concurrently conducted, and the results consistently corroborated our previous findings (Figure 4G,H). These data demonstrated that the MMP10 was assuredly upregulated.

To confirm the ability of MMP10 to promote VSMC calcification, we conducted in vitro stimulation experiments by treating VSMCs directly with MMP10 active protein. After graded stimulation with MMP10, VSMCs exhibited varying degrees of calcification which indicating a dose‒response relationship (Figure 4I,J). These results jointly manifested that TRIM35 knockout in ECs increased MMP10 expression and secretion, which subsequently induced VSMC calcification.

### 3.5 TRIM35 Induces the Ubiquitination of RelB K63 without Affecting Protein Stability or Degradation

**Figure 5:**
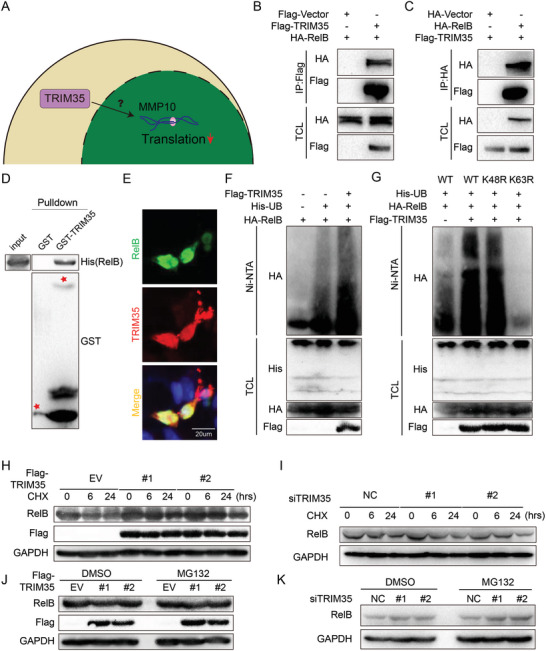
TRIM35 Induces the Ubiquitination of RelB K63 without Affecting Protein Stability or Degradation. A) Schematic for exploring potential binding protein regulating TRIM35‐mediated MMP10 expression. B,C) HUVEC were transfected with indicated plasmids, followed by inmunoprecipitation (IP) and Western‐blotting. D) Purified GST‐TRIM35 fusion protein was incubated with cell lysate containing His‐RelB and GST‐beads, followed by Western‐blotting. E) IF staining for TRIM35 (red), RelB (green) and DAPI (blue) in HUVEC. F) HUVEC were transfected with Flag‐TRIM35, HA‐RelB and His‐UB plasmids, followed by in vivo ubiquitination assay and Western‐blotting. G) HUVEC were transfected with Flag‐TRIM35, HA‐RelB (wild‐type, K48R or K63R) and His‐UB plasmids as indicated, followed by in vivo ubiquitination assay and Western‐blotting. H,I) TRIM35‐overexpressing HUVEC cell lines (#1, #2) in H and TRIM35 knockdown HUVEC cell lines with siRNA in I treated with CHX (100 ug mL^−1^) for 0h, 6h, 24h, followed by Western‐blotting. J,K) TRIM35‐overexpressing HUVEC cell lines (#1, #2) in J and TRIM35 knockdown HUVEC cell lines with siRNA in K treated with DMSO or MG132 for 5 h respectively, followed by Western‐blotting.

After knowing that endothelial TRIM35 knockout could induce MMP10 secretion and accompanying VSMC calcification, we sought to investigate the inner molecular mechanism how TRIM35 regulates MMP10 expression and secretion. We examined the TRIM35‐binding proteins reported previously.^[^ ^44^ ^]^ Interestingly, we did not observe direct binding between TRIM35 and MMP10, suggesting that MMP10 may not be a substrate for ubiquitination by TRIM35.^[^ ^44^ ^]^ However among the potential substrates of TRIM35, we identified RelB, a transcription factor involved in the noncanonical NF‐κB signaling pathway known to regulate cell migration and tissue regeneration.^[^ ^45^ ^]^ MMP10 expression is regulated by the NF‐κB pathway,^[^ ^46^ ^]^ but the role of noncanonical pathways in regulating MMP10 expression in tissue repair has not been fully elucidated. Hence, we assumed that TRIM35 modulated the expression of MMP10 by ubiquitinating RelB (**Figure** **5**A). To validate the interaction between TRIM35 and RelB, we overexpressed Flag‐TRIM35 and HA‐RelB in HUVECs. Co‐immunoprecipitation assay validated the interaction between TRIM35 and RelB (Figure 5B,C). Furthermore, it also been confirmed though GST pulldown using recombinant GST‐TRIM35 protein and His‐RelB (Figure 5D). Immunofluorescence demonstrated substantial overlap between TRIM35 and RelB in HUVECs nucleus (Figure 5E). These results validated TRIM35 interacted with RelB.

As TRIM35 is an E3 ligase, to examine whether TRIM35 ubiquitinates RelB, we co‐transfected Flag‐TRIM35, His‐ubiquitin (Ub), and HA‐RelB into HUVECs and founding that TRIM35 promoted the ubiquitination of RelB (Figure 5F). TRIM35 has been reported to mediate both K48‐ and K63‐linked ubiquitination,^[^ ^29^ ^]^ therefore, we performed in vitro ubiquitination assays with the WT, K48R, or K63R ubiquitin mutant to determine the type of TRIM35‐induced RelB ubiquitination. Transfection with the K48R mutant did not significantly reduce RelB ubiquitination, but the K63R mutant did (Figure 5G). This finding suggested that TRIM35 catalyzed the formation of K63 ubiquitin chains rather than K48 ubiquitin chains on RelB. Moreover, to assess the impact of TRIM35 ubiquitination on the stability and degradation rate of RelB, we established TRIM35 KO HUVEC cells by CRISPR‐Cas9 editing and verified KO efficacy by T7 exonulease digestion (Figure S10, Supporting Information). Overexpression or knockdown of TRIM35 did not affect the abundance of RelB in HUVEC treated with cycloheximide or MG132 indicating that the ubiquitination of RelB (Figure 5H–K). These data accurately pinpointed TRIM35 interacted with RelB to mediate K63‐linked ubiquitination, which was independent of protein degradation via the ubiquitin‒proteasome pathway.

### 3.6 TRIM35 Promotes the Noncanonical NF‐κB Signaling Pathway through K63‐Ubiquitinated RELB at K242/K327 and Regulates MMP10 Expression

**Figure 6:**
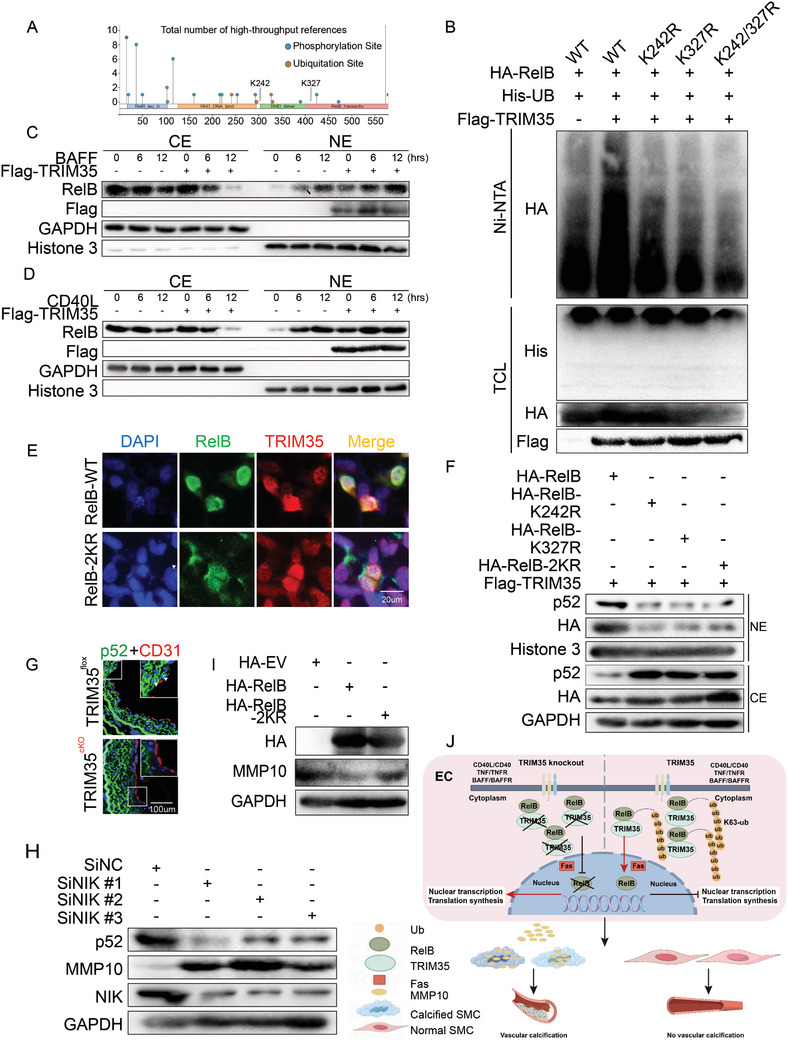
TRIM35 Promotes the Noncanonical NF‐κB Signaling Pathway through K63‐ubiquitinated RelB at K242/K327 and Regulates MMP10 Expression. A) Potential ubiquitination sites of RelB predicted by phosphositePlus. B) HUVEC were transfected with Flag‐TRIM35, HA‐RelB (wild‐type, K242R, K327R and K242/327R) and His‐UB plasmids as indicated, followed by in vivo ubiquitination assay and Western‐blotting. C,D) HUVEC were transfected with plasmids as indicated and treated with BAFF (50ng mL^−1^) (C) or CD40L (0.1µg mL^−1^) (D) for 0h, 6h, 12h, followed by cytoplasmic‐nuclear fractionation assay. E) HUVEC were transfected with plasmids as indicated and followed by ICC for TRIM35 (red), RelB (green) and DAPI (blue). F) HUVEC were transfected with plasmids as indicated and followed by cytoplasmic‐nuclear fractionation assay. G) IF staining for p52 (green) and CD31 (red) in isografts from TRIM35flox or TRIM35cKO mice. H) HUVEC were transfected with negative control (siNC) or siNIK (#1, #2, #3). I)EC isolated from TRIM35cKO were transfected with HA‐empty vector (EV), HA‐RelB (wild‐type), and HA‐RelB‐2KR, followed by detection with indicated antibodies. J) Schematic for endothelial TRIM35 protect grafts against calcification via ubiquitinating RelB to regulate MMP10 synthesis and secretion.

Next, in order to identify the specific mechanism of RelB‐K63 ubiquitination, we first sought to investigate the RelB ubiquitination sites induced by TRIM35. By utilizing post‐translational modification site database (PhosphoSitePlus) to analyze potential ubiquitination sites on RelB, we screened and revealed two potential sites for TRIM35‐promoted ubiquitination of RelB: lysine 242 and 327 (K242 and K327) (**Figure** **6**A). Then we mutated these two candidate sites of RelB to arginine (K to R) and compared the ubiquitination level of wild‐type RelB with the mutants. Through in vitro ubiquitination assay, we confirmed that the mutation of both K242R and K327R could tremendously abrogated the TRIM35‐induced RelB ubiquitination, which suggesting TRIM35 ubiquitinated RelB at its K242 and K327 sites (Figure 6B).

Given that K63 ubiquitination normally regulates protein function^[^ ^47^ ^]^ and RelB primarily exerts its biological functions within the nucleus,^[^ ^48^ ^]^ we secondly attempted to identify the effect of TRIM35 on the cellular localization of RelB. HUVEC was treated with BAFF or CD40L (both stimulants of the noncanonical NF‐κB signaling pathway) and performed cytoplasmic‐nuclear fractionation assay to assess the cellular localization of RelB. The overexpression of TRIM35 could significantly promoted the stabilization of nuclear localization of RelB (Figure 6C,D). Then, we further measured the effect of RelB ubiquitination on its nuclear localization. Fluorescence co‐localization (Figure 6E) and cytoplasmic‐nuclear fractionation assay (Figure 6F) similarly illustrated the reduced location in nucleus of both RelB K242R and K327R, suggesting that TRIM35 could maintain RelB nuclear localization through ubiquitination at K242 and K327. Consistently, we also find the decreased nuclear location of p52 in TRIM35cKO isografts upon (Figure 6G). p52 is a key regulator of NF‐κB signaling pathway, activation of noncanonical NF‐κB signaling pathway requires p52 nuclear localization and dimerized with RelB.^[^ ^48^, ^49^ ^]^ Together, these results indicated that TRIM35‐induced K63‐ubiquitination at RelB K242/K327 maintained its nuclear localization and activated the noncanonical NF‐κB signaling.

RelB has been revealed currently to act as both activator and inhibitor on noncanonical NF‐κB‐dependent gene transcription.^[^ ^50^, ^51^ ^]^ Thus, sequentially to further investigate the effect of RelB contributed to MMP10 transcription, we inhibited the noncanonical NF‐κB pathway by transfecting siRNA targeting NF‐κB‐inducing kinase (NIK), a necessary protein for noncanonical NF‐κB pathway activation.^[^ ^48^ ^]^ We observed that the inhibition of the noncanonical NF‐κB pathway was accompanied by an increase in MMP10 (Figure 6H). Furthermore, we parallelly checked the effect of TRIM35‐induced RelB ubiquitination on MMP10 expression. Overexpression of RelB in TRIM35 knockout EC remarkably decreased MMP10 expression, however, overexpression of RelB‐2KR mutant could partly restored it (Figure 6I). Taken together, these findings confirmed that TRIM35 K63‐ubiquitinated RelB at K242/K327 and enhanced the activation of the noncanonical NF‐κB pathway, which subsequently inhibited MMP10 secretion and VSMC calcification (Figure 6J).

### 3.7 Inhibiting the Physiological Function of MMP10 Effectively Alleviates Vascular Calcification

**Figure 7:**
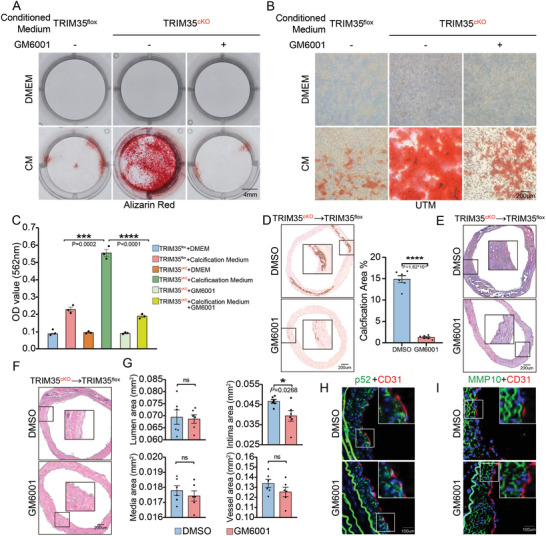
Inhibiting the Physiological Function of MMP10 Effectively Alleviates Vascular Calcification. A‐C) Alizarin Red (A) staining for calcium nodules in pVSMC treated with DMSO or GM6001 (10uM) indirectly co‐cultured with TRIM35flox or TRIM35cKO pEC. UTM was shown in B. Quantification of Alizarin Red staining was shown in C, *n* = 3. D‐G) Von Kossa and Nuclear Fast Red staining (D), Masson staining (E) and HE staining (F and G) of isograft arteries from 8W TRIM35cKO mice locally administered GM6001 or DMSO, *n* = 6 in each group. H,I) IF staining for p52 (green, in H), MMP10 (green, in I) and CD31 (red) in isograft arteries from 8W TRIM35cKO mice locally administered GM6001 or DMSO. Data are means and SEM, *p < 0.05, **p < 0.01, ***p < 0.001, ****p < 0.0001.

After identifying the mechanism of TRIM35 prevent isograft calcification by inhibiting MMP10 secretion, we sequentially used GM6001 (a broad‐spectrum MMP protein inhibitor) to validate potential targeting strategies for MMP10. By treating VSMC‐TRIM35cKO coculture model with GM6001 we observed a significant reduction in calcium deposition (**Figure** **7**A–C). In situ blocking MMP10 activity on isografts also significantly alleviated calcification and collagen deposition in the TRIM35cKO transplantation model (Figure 7D,E). HE staining revealed evidently a decrease of thickened neointima through MMP10 inhibition (Figure 7F,G). To validate the exclusive function of MMP10 among MMPs family members, MMP10 neutralizing antibody was also applied in situ after CABG. Similarly, calcification remission was detected in TRIM35 cKO isografts (Figure S11, Supporting Information), which was reasonable to suggest blocking MMP10 activity could prevent against vascular calcification.

At last, we checked the activation of the noncanonical NF‐κB pathway and the expression of MMP10 after GM6001 stimulation. There was no significant difference in the accumulation of p52 and MMP10, showing that the remittence of vascular calcification was indeed driven by blocking biological function of MMP10 rather than its expression, or noncanonical NF‐κB pathway activation (Figure 7H,I). Besides, we also found that GM6001 alleviated VSMC phenotypic transition mediated by TRIM35cKO‐induced calcification (Figure S12, Supporting Information), which complemented evidences for vascular calcification therapeutic medication of targeting MMP10. These results above collectively proved that targeting MMP10 could be a novel therapeutic approach for protecting isograft arteries against vascular calcification.

### 3.8 Plasma MMP10 Predicts Unfavorable Prognosis in CABG Patients

**Figure 8:**
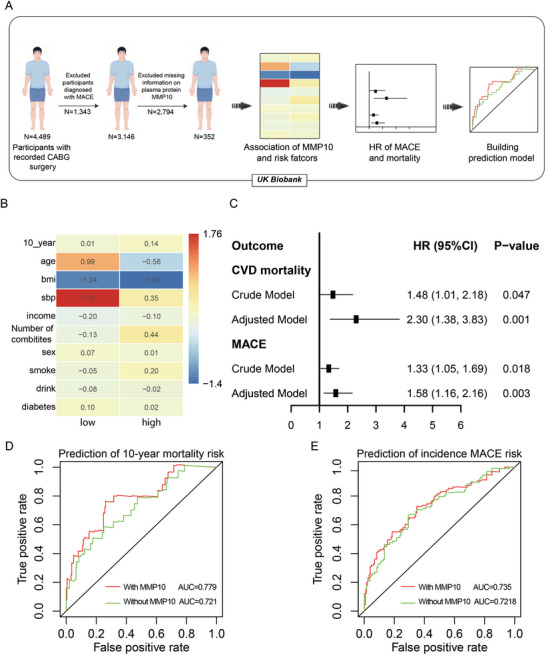
Plasma MMP10 Predicts Unfavorable Prognosis in CABG Patients. A) Schematic for analysis flowchart. B) Heatmaps showing MMP10 was highly associated with risk factors of adverse outcomes in CABG patients. C) Association between risk of CVD mortality, incident MACE and MMP10 in crude model or adjusted model. D,E) ROC curve predicted the 10‐year mortality risk and incidence MACE risk prediction of MMP10.

After determining that endothelial MMP10 secretion was responsible for SMC aberrant calcium deposition and vascular calcification, we also wondered whether plasma MMP10 level reflected the incidence of restenosis or mortality after CABG. For this purpose, we analyzed the plasma MMP10 level of CABG patients from the UK Biobank,^[^ ^52^ ^]^ a prospective cohort study previously described in detail recruited more than 500 000 adults aged 40 to 69 years living in the United Kingdom between 2006 and 2010 (**Figure** **8**A and Table S1 Supporting Information). It was heartening to note that the plasma MMP10 concentration was strongly associated with risk factors for adverse outcomes in CABG patients, as shown in the heatmap (Figure 8B). Furthermore, according to both the crude model and the fully adjusted model, MMP10 was positively associated with CVD mortality and incident major adverse cardiovascular events (MACE) (Figure 8C), suggesting that higher MMP10 levels tend to predict the occurrence of MACE and CVD death. Moreover, we applied receiver operating characteristic (ROC) curve analysis to further evaluate the ability of MMP10 to predict mortality risk, the results of which additionally supported our hypothesis. The inclusion of MMP10 substantially enhances the area under the AUC curve in comparison to adjustments made solely for the covariates depicted in the heatmap (Figure 8D–E). Besides, to further reveal the stronger link between MMP10 and calcification events, we also used data from FHS (Framingham Heart Study) examinations 7 and exploring the high levels of MMP10 were associated with higher TAC scores of CABG patients, which was an important evaluation of measuring coronary calcification. (Figure S15 and Table S2, Supporting Information). All that evidences showed that human plasma MMP10 was associated with MACE incidence and CVD mortality of CABG patients, which might become a strong predictor of undesirable prognosis.

## 4 Discussion

A number of clinical studies have confirmed that severe lesion calcification after CABG is associated with increased mortality, as shown by 5‐year outcome data from the Synergy Between PCI With Taxus and Cardiac Surgery (SYNTAX) trial and SYNTAX registry,^[^ ^1^ ^]^ indicating that calcification is a strong indicator of poor prognosis after CABG revascularization, in addition to reflecting the risk of atherosclerosis.^[^ ^53^ ^]^ In a previous study, a histopathological of the human bypass artery showed that extensive calcification in the coronary artery occurred not only in restenosis segments but also in adjacent proximal and distal positions because it was induced by diffuse coronary atherosclerotic disease.^[^ ^54^ ^]^ Similarly, in our study, we found isograft vascular calcification in CABG showed obvious characters of specific aggregated in stenosis region and was induced by inflammation signaling imbalance and EC‐driven MMP10 release. Using isograft mouse model, calcified segments developed proximal to the anastomosis, similar to what has been observed in graft atherosclerosis^[^ ^22^ ^]^ which is common in human artery grafts.^[^ ^55^, ^56^ ^]^ In general, our findings delineated and exposited the pathological changes of vascular calcification after CABG was comprehensive and profound enough to characterize the similar lesion that occurs in the advance stage of human graft atherosclerosis.

Another intriguing finding of this study is we exhibited the unique regulation of EC for SMC calcium deposition. Although EC‐SMC interaction was considered to be a key factor in many vascular diseases, especially for vascular remodeling, current studies usually focus on the paracrine function of infiltrated monocyte/macrophages in regulating SMC phenotypic change. monocyte/macrophages may release TNF‐α and activate osteogenic program of SMCs via Msx2‐Wnt signaling.^[^ ^57^ ^]^ Receptor activator of NF‐κB ligand (RanKL) also can promote osteochondrogenic differentiation and calcification of SMC, by increasing pro‐calcific cytokines IL‐6.^[^ ^58^ ^]^ Besides, the secretion of IL‐1β by macrophage was similarly involved in calcification progression.^[^ ^38^ ^]^ Of note, we pioneeringly found that EC‐SMC interaction also revealed great impact during vascular calcification. Endothelial TRIM35 may interact with SMC as a mineralization inhibitor, lack of TRIM35 lead to MMP10 expression and secretion upregulated and spontaneous SMC calcification, which has never been focus on.

The vascular calcification hypothesis is controversial. Blood stasis and low shear stress resulting from competitive flow between native and bypass grafts may be the underlying mechanism of greater calcification in grafted native arteries.^[^ ^55^, ^59^ ^]^ Although multiple mechanisms are involved during the pathogenesis of vascular calcification, little is known about the TRIM35 ubiquitination‐mediated regulation of the noncanonical NF‐κB signaling pathway. Different types of ubiquitin chains covalently attached to target proteins have emerged as important regulators of protein functions. K48‐linked polyubiquitination is a signal for the proteasomal degradation of target proteins, whereas K63‐linked polyubiquitination is a nonproteolytic mode of modification that is important for the assembly and function of protein signaling complexes.^[^ ^60^, ^61^ ^]^ Like most TRIM proteins, TRIM35 contains an N‐terminal RING domain and can function as an E3 ubiquitin ligase. TRIM35 is ubiquitously expressed in different tissues and was initially implicated as playing a role in apoptosis.^[^ ^62^ ^]^ Regarding its role in innate immunity, TRIM35 has been reported to negatively regulate toll‐like receptor 7/9 (TLR7/9)‐mediated type I IFN production by inducing interferon regulatory factor 7 (IRF7) degradation.^[^ ^63^ ^]^ Intriguingly, TRIM35 has two ubiquitination functions in the antiviral immune response to IAV infection: the K63‐linked polyubiquitination of TRAF3 promotes the activation of the RIG‐I antiviral signaling cascade and the production of type I IFN; and K48‐linked polyubiquitination of viral PB2 leads to the direct proteasomal degradation of PB2, an essential component of the vRNP complex that is responsible for the transcription and replication of the IAV genome, and counteracts the PB2‐mediated suppression of the K63‐linked polyubiquitination of TRAF3. Li et al. reported that TRIM35 is a positive regulator of RIG‐I‐mediated innate immunity, leading to the enhanced production of type I IFNs by facilitating the K63‐linked polyubiquitination of TRAF3.^[^ ^64^ ^]^ However, our study revealed an unprecedented function of TRIM35, i.e., a distinct ability to modify vascular calcification after CABG. Endothelial TRIM35 was highly expressed in artery grafts, but SMC calcification in artery grafts progressed substantially when TRIM35 was conditionally knocked out both in vitro and in vivo. Meanwhile, we discovered for the first time that RelB was constitutively ubiquitinated in endothelial cells. Mechanistically, endothelial TRIM35 can directly K63‐ubiquitinate RelB at K242/K327 and induce nuclear translocation, resulting in the activation of the noncanonical NF‐κB pathway and the inhibition of MMP10 transcription.

MMP10, also known as matrix metalloproteinase 10 or stromelysin 2, is a protease encoded by the human MMP10 gene.^[^ ^39^ ^]^ MMP10 is mainly involved in the degradation of extracellular matrix, especially proteoglycans and fibronectin.^[^ ^40^ ^]^ Although it plays an important role in various physiological and pathological processes including tissue remodeling and repair, cell migration, inflammatory response, and tumor progression,^[^ ^41^, ^42^ ^]^ few studies have addressed the potential relationship between MMP10 and vascular calcification. To explore the mechanism of VSMC calcification driven by MMP10, in this study, we identified the top six relevant cytokine‐related genes after RNA sequencing screening. These genes included MMP10 and MMP16, both of which are members of the MMP family. Since previous studies have suggested that MMP10 is a secretory protein, while MMP16 is usually regarded as a transmembrane protein with weak exocrine ability.^[^ ^41^ ^]^ We further hypothesized that endothelial TRIM35 depletion causes smooth muscle cell epigenic switching and calcification in a paracrine manner, and eventually confirmed transplant vascular calcification was induced by MMP10 secreted by endothelial TRIM35 knockdown.

Clinical molecular diagnosis aims to delineate important biological disease development, prognosis, and outcomes as an important supplement to traditional diagnostic methods.^[^ ^65^ ^]^ Multiple clinical trials have revealed the relationship between MMP10 and adverse cardiovascular disease outcomes. During the acute phase, the inflammatory response could stabilize myocardial infarction and atherogenic vascular impairment, and excessive inflammation may cause injury to the nearby myocardium and vascular wall and increase the risk of revascularization after CABG.^[^ ^66^ ^]^ Ripplinger and Kessinger et al. serially assessed thrombus inflammation and the effects of MMP activating agents on vascular stenosis. The extent of macrophage inflammation and MMP activation on day 4 strongly predicted the extent of thrombus reduction (area) on day 6 (*p* < 0.05).^[^ ^67^ ^]^ When testing the “inflammatory hypothesis” in clinical trials of anti‐inflammatory therapeutics, plasma MMP‐10, MCP1, and IL8 levels were markedly reduced and high signal levels were reduced in vascular segments with restenosis.^[^ ^68^, ^69^ ^]^ This evidence strongly supports the potential of MMP10 as a diagnostic and predictive marker for adverse cardiovascular events. We also found that the plasma MMP10 level could predict restenosis incidence and mortality after CABG. MMP10 was strongly associated with CVD mortality and incident MACE risk, as the hazard ratio (HR) for CVD mortality risk was 2.30 (95% CI, 1.38‐3.83) and that for MACE risk was 1.58 (95% CI, 1.16–2.16). Therefore, a higher MMP10 level may be a powerful factor for predicting poor outcomes of CABG patients.

## 5 Conclusion

In summary, our study sheds light on characteristic changes of SMC injury and calcification during vascular remodeling after CABG. EC‐SMC interaction‐driven phenotype switching and aberrant calcium deposition are the essential factors of vascular calcification. The regulation mechanism of endothelial TRIM35 inhibiting vascular calcification during arterial isograft remodeling is driven by the noncanonical NF‐κB signaling pathway activation through K63‐ubiquitinated RelB at K242/K327, resulting intrinsic inhibition of endothelial MMP10. We lastly verified that plasma MMP10 may be a strong predictor of undesirable prognosis and MACE in CABG patients. Conclusively, targeting MMP10 could be a potential therapeutic strategy for treating vascular calcification after CABG.

## 6 Experimental Section

### Animal Experiments

H11‐Cdh5‐iCre mice (Strain NO. T004712) and TRIM35flox mice (Strain NO. T005401) were purchased from GemPharmatech (Nanjing, China). 8‐week‐old C57BL/6J were obtained from Hunan SJA Laboratory Animal Co., Ltd (Hunan, China). TRIM35 conditional endothelial KO mice (TRIM35^cKO^) were generated by Cdh5‐iCre and TRIM35^flox^ mice cross breeding. The genotyping of H11‐Cdh5‐iCre, TRIM35^flox^, and TRIM35^cKO^ mice was verified by PCR and histology staining. All mice were housed in specific pathogen free (SPF) facility, and fed with a standard chow diet in experiencing standard temperature, humidity, and light‐dark cycle (12h).

The procedure of isograft transplantation was conducted as previously described. In summary, donor aortic segments were collected from either TRIM35flox mice or TRIM35cKO mice, washed, and suspended in a saline solution supplemented with 100 U mL^−1^ heparin. A midline incision was made on the ventral side of the neck, followed by resection of the right sternocleidomastoid muscle. The right common carotid artery was then isolated and immobilized by clamping at both distal and proximal ends. After dissecting the artery in the middle, artery cuffs were placed at both distal and proximal ends. Subsequently, the aortic segments from the donor mice were implanted by sleeving over the artery cuffs between the two ends of the recipient carotid artery of TRIM35flox mice, all of which were of C57BL/6J background. None of the mice selected for surgery had undergone any prior treatments or procedures. Sample sizes were determined based on previous experience with similar experiments.

For en face vascular staining, the grafted donor aortic segments were harvested under a dissecting microscope and the connective tissues around the aorta were removed. Incised the aorta longitudinally to expose the intima thoroughly and avoided connecting with the intima. Fixed with 4% paraformaldehyde overnight and soaked in glycine for 30 min. Then we blocked the aortic fragments with 5% BSA for 1 h and stained with CD31 antibody for proper time. The en face sections were photographed by fluorescence microscope after sealing.

For Evan's blue staining, we injected 2% Evan's Blue solution (40mg/kg) into the mice tail vein then sacrificed the mice after 30 min. Mice were then sequentially perfused with prechilled PBS solution and 4% paraformaldehyde until the lungs and livers became pale. After harvesting, the graft aortas were photographed by a digital camera.

The sample size for animal experiments was predetermined with α value of 0.05 and 1‐β value of 0.8 based on our preliminary experiments. Each experiment was repeated for at least three times. All animal experiments and protocols were conducted in compliance with the guidelines of the Animal Care and Use Committee of Central South University (2021‐XMSB‐0070).

### Human Samples

All the human specimens used have obtained informed consent from the patients. Aortic samples from atherosclerotic and non‐atherosclerotic sites were collected from 7 patients undergoing CABG surgery at Xiangya Hospital. mRNA and protein were extracted immediately after obtaining the tissues, and a portion of the vessels was taken for histological experiments. The human tissues involved in this study were approved by the Medical Ethics Committee of Third Xiangya Hospital, Central South University (I 20006).

### Cell Lines and Cell Experiments

HEK293FT and 293A were obtained from the American Type Culture Collection. Human smooth muscle cells (SMC, purchased from Otwo Biotech) were cultured in Dulbecco's Modified Eagle Medium (DMEM, Gibco) containing 10% FBS (Gibco). Human umbilical vein endothelial cells (HUVEC, purchased from National Infrastructure of Cell Line Resource) were cultured in endothelial cell culture medium (ECM, SCIENCELL).

For conditional medium extraction, primary endothelial cells were cultured with ECM and inoculated into 6‐well plates at a density of 1 × 10^6^ mL^−1^. On the third day of inoculation, when the cell density reached 90%, the cells were replaced with DMEM without FBS, incubated at 37°C in 5% CO2 cell culture chamber, extracted conditioned medium 24 hours later, centrifuged at 3000 rpm for 10 minutes, collected supernatant and stored at −20°C.

### Primary Cell Isolation and Culture

For primary mice endothelial cells isolation, ether anesthesia of male 12‐week‐old TRIM35^flox^ or TRIM35^cKO^ mice and perfused into the heart's right ventricle to flush out accumulated blood with prechilled sterile PBS solution until the lungs and livers became pale. The lungs were removed and transferred to a 6‐well plate for further digestion. The tissue was minced in a sterile, clean cell culture dish with pre‐sterilized scissors until the tissue became mushy. Resuspended the tissue with pre‐warmed collagenase solution and transferred to a 15 mL conical tube. Shaked on a rotating mixer for 1 h at 37 °C, 220 rpm. Filtered the suspension through a 100 µm cell strainer into a fresh 50 mL conical tube and washed the cell strainer with 2 mL DMEM to stop digestion and wash down residual cells. Centrifuged the cell suspension at 400 g, 4 °C for 8 min, removed the supernatant, resuspended the deposit with 500 µL DMEM and transferred the suspension into a 1.5 mL conical tube. Anti‐CD31 immunobeads were then added to the suspension and shaken the suspension on a rotating mixer at 4 °C overnight. Endothelial cells attached to the magnetic beads were isolated by a magnetic separation rack and seeded into a gelatin‐coated 24‐well plate, changing ECM every second day. The primary cells passaged when the cells reached 80% to 90% confluency. Secondary separation performed the same as described above by anti‐CD31 immunobeads but using anti‐CD102 immunobeads.

For primary mice vascular smooth muscle cells isolation, we conventionally anesthetized mice and perfused PBS solution into the heart's right ventricle as described above. The aorta was separated, and the extravascular tissue was removed until the aorta became transparent. The digestion procedure was consistent with the isolation of primary endothelial cells mentioned above, except for the digestion time, which increased to 4 h. DMEM was added to the cell suspension to end digestion. Anti‐αSMA immunobeads were then added to the suspension and shaken the suspension on a rotating mixer at 4 °C overnight. Cells attached to the magnetic beads were then isolated by a magnetic separation rack and resuspended cells with 1 mL DMEM containing 10% FBS, and seeded in a 12‐well plate, changing the culture medium every second day.

### Quantitative Real‐Time Polymerase Chain Reaction (qRT‐PCR)

Total RNA was extracted by TRIzol reagent (Invitrogen, CA, USA). Equal 1µg RNA was used as template for reverse transcription. SYBR qPCR Master Mix (Vazyme, Nanjing, China) was used according to the manufacturer's instructions. All amplification reactions were carried out over 40 cycles. Each sample was performed in triplicate and the data were analyzed by ΔΔCT method. All samples were normalized to GAPDH.

### Immunoblot Analysis and Immunoprecipitation

All cell samples were lysed in RIPA buffer (Beyotime, Shanghai, China) supplemented with protease inhibitors cocktail (Roche) to extract whole cell protein. The cell lysates were centrifuged at 12 000 g, 4 °C for 15 min and the protein concentrations were measured by a BCA Protein Assay Kit (Thermo Scientific). Equal amounts of total protein were resolved on 8%‐12% SDS‐PAGE gels and transferred onto a PVDF membrane. Then the membrane was blocked by 5% BCA in PBS solution and incubated with primary antibodies at 4 °C overnight and secondary antibodies for 1 hour. The protein bands were visualized by Immobilon Western HRP Substrate Kit (Millipore).

For immunoprecipitation experiments, the cell lysates were incubated with anti‐Flag conjugated magnetic beads or anti‐HA conjugated magnetic beads overnight at 4 °C. After incubation, the precipitates were washed by IP lysis buffer (50mM Tris pH 7.5, 150mM NaCl, 1mM EDTA, 0.1% NP‐40) three times followed by suspending in IP lysis buffer. After denaturation at 100 °C for 5 min, the samples were analyzed by western blotting.

### Expression and Purification of Recombinant Proteins

*E. coli* BL‐21 was used to express GST‐fusion protein contained in the pGEX‐6p3 vector containing TRIM35. LB medium with 0.1 mM IPTG (isopropyl 1‐thio‐β‐D‐galactopyranoside, Sigma) was used to culture transformed bacteria expressing recombinant GST‐TRIM35 at 25 °C for 4 h. After bacterial cultivation and amplification, the bacteria were centrifuged and lysed. Glutathione beads were used to incubate bacterial lysate overnight at 4 °C then washed three times. Protein concentration was measured by Coomassie stain. Flag‐tagged TRIM35 was transiently transfected into 293FT cells for expression and purification. For purification, the cell lysates collected from 293FT 2 days later after transfection was incubated with anti‐Flag beads overnight at 4 °C. Purified TRIM35 proteins were eluted using 3 × Flag peptides (Sangon Biotech) and employed for relevant experiments.

### GST‐Pull Down Assay

The purified recombinant GST‐TRIM35 protein was used for in vitro GST‐pull down assay. His‐RelB was overexpressed in 293FT for 2 days. Cell lysates were incubated with His‐tag beads in 4°C overnight for His‐RelB enrichment. His‐tag beads were then incubated with recombinant GST‐TRIM35 protein at room temperature for 1h. Samples were analyzed by immunoblotting.

### Ubiquitination Assay

293FT cells were enzymatically digested and seeded in culture dishes at a density of 40%‐50%. After the cells adhered to the dish, plasmids were transfected. After 48 h, MG132 was added for a 4‐hour treatment, followed by PBS washes twice. Buffer C was added, and the cells were sonicated for extraction of whole‐cell lysates. Ni‐NTA agarose beads were added to whole‐cell lysates and rotated at 4°C overnight. The supernatant was then discarded after centrifugation, and the beads were washed twice sequentially with Buffer C, Buffer D, and Buffer E. Finally, the beads were resuspended in a sample buffer containing imidazole and heated to denature the proteins. Western blot was performed immediately.

### HE and Masson's Trichrome Staining

The frozen sections were thawed to room temperature and fixed with 4% polyformaldehyde for 30 min. The sections were immersed in a series of gradually increasing ethanol concentrations for dehydration. Hematoxylin and Eosin (ZSGB‐BIO, Beijing, China) were used for staining tissue sections. After staining, the sections were dehydrated with ethanol, dried in an incubator, and then mounted.

Masson's Trichrome staining was applied by Masson trichrome staining Kit (Abiowell, Changsha, China) according to the manufacturer's protocol.

### Alizarin Red and Von Kossa Staining

The experimental procedure for Alizarin Red staining and Von Kossa staining involved de‐fatting and rehydration of fixed tissue samples, followed by applying Alizarin Red dye solution (Abiowell) to tissue sections. Subsequently, the sections were rinsed with distilled water, gradually dehydrated, and finally cleared with a clearing agent before being coverslipped. The results revealed calcium deposits in the form of red or orange‐red staining visible under an optical microscope.

For Von Kossa staining, in simple terms, the tissue sections were soaked in 5% silver nitrate buffer, exposed to ultraviolet light for 30 min, rinsed with deionized water, incubated with 5% sodium thiosulfate, and then exposed to rapid nuclear red staining. Calcium crystals were stained brown, the cytoplasm was stained pink, and the cell nuclei were stained red.

### Immunofluorescence and Immunohistochemical Staining

For sample collection, we harvested mice aorta after transplantation and washed the tissues in PBS solution three times, then fixed in 4% polyformaldehyde overnight. Tissues were dehydrated in 30% sucrose solution until settled to the bottom of the tube. After dehydration, tissues were embedded in OCT and stored at ‐80 °C. Tissues were then sliced into 8–10 µm sections. Frozen sections were thawed in advance, recovered to room temperature in PBS, and OCT was washed away before staining. Subsequently, 5% BSA (Songon Biotech) was used for blocking for 30 min. For immunofluorescence, after dilution, the primary antibody was incubated at 4 °C overnight. The following day, secondary antibody and DAPI were sequentially applied, and the slides were sealed using an anti‐fade mounting medium.

For immunohistochemical, DAB staining solution was used according to the manufacturer's protocol, and Hematoxylin staining was used for an appropriate time.

### Quantification of SMC Calcification

SMC was exposed to a calcifying medium consisting of 3 mM calcium chloride (Solarbio) and 10 mmol L^−1^ beta‐glycerophosphate (Solarbio) to promote the formation of calcification. The calcification status of SMC was demonstrated through Alizarin Red staining. Red or orange‐red staining indicated a positive result. Calcification level was calculated by measuring O.D value (405 nm) with a spectrophotometer.

### siRNA Sequence and Transfection

Small interfering RNA (siRNA) against human TRIM35, human NIK, and scramble siRNA were designed and synthesized by RiboBio (Guangzhou, China). siRNA (50 nM) transfection in HUVEC was applied by LipoRNAi (Beyotime, Changsha, China) reagent according to the manufacturer's protocol. The siRNA sequence used in the study were 5’‐CTGGAAGAAGATGCTTGCA‐3’ (human TRIM35 #1), 5’‐GCGAGCTGTCTTTCTATGA‐3’ (human TRIM35 #2), 5’‐GAAGATGAAGCAGCTCACA‐3’ (human TRIM35 #3), 5’‐CGCCAAAUCAAGCCAAUUAdTdT‐3’ (human NIK #1), 5’‐GUGAGAAGAACCCAUCAAAdTdT‐3’ (human NIK #2), 5’‐GCCAGUCCGAGAGUCUUGAUCAGAUdTdT‐3’ (human NIK #3), 5’‐UUCUCCGAACGUGUCACGUTT‐3’ (scramble siRNA).

### Alkaline Phosphatase Activity Assay

The alkaline phosphatase (ALP) activity of SMC stimulated with calcification medium or control medium was assessed using an ALP assay kit (Beyotime). In simple terms, the ALP (alkaline phosphatase) activity assay procedure involved sample preparation, preparation of the reaction mixture, setting reaction conditions, conducting the enzyme reaction, measuring absorbance, and finally calculating ALP activity. In this experiment, ALP enzymes in the samples converted substrates into products, and their absorbance was used to quantify ALP activity.

### Bulk RNA Sequencing

Bulk RNA sequencing was performed on primary endothelial cells isolated from male 12‐week‐old TRIM35^flox^ or TRIM35^cKO^ mice. RNA isolation was performed with TRIzol reagent, as described above. A strand‐specific cDNA library was constructed, and sequencing was performed on the Illumina HiSeq 2000/4000 platform. (Novogene Bioinformatic Technology, Beijing, China). All subsequent analyses were conducted using clean data.

### Data Analysis of Singel‐Cell RNA‐Sequencing

Cell Ranger (V.3.1.0) and R package Seurat version (V.4.0.3) were used for single‐cell RNA‐sequencing (scRNA‐seq) analysis. In simple terms, we generated a normalized gene expression matrix harboring 16342, 11459, and 11986 cells for sham, 2‐week, and 4‐week transplant WT mouse models. To validate our results, we empirically introduced well‐defined markers to explore cell types from cell clusters that included Pecam1, Egfl7, Cdh5, Cldn5, and Cytl1 for EC cells; Acta2, Tagln, Myl9, Cald1, Myh11, Cnn1, Mylk4, and Col1a1 for SMCs. s; Adamtsl1, Lamc3, and Sfrp2 for fibroblast cells; and Fcer1g, Cxcl2, Cd74, C1qb, C1qc, Lgals3, and Cd68 for both immune and inflammation cells. Gene set enrichment analyses and ligand‐receptor analysis were performed. ligand‐receptor pairs between cell types following a similar procedure as previously described. Genes with log2 fold change >0.5, and values of *p* < 0.05 were considered differentially expressed. Pathway enrichment analysis for group comparison was performed with R package ReactomeGSA34 using the analyze_sc_clusters function.

### Data Analysis of Endothelial Cell‐Smooth Muscle Cell Communication

To infer and analyze cell‐to‐cell communication, CellChat(1.5.0) was used, an R package that enables quantitative inference, visualization, and analysis of cell‐to‐cell communication networks from single‐cell data. By integrating KEGG databases and literature, CellChat constructed cellchatDB, a cell communication database including public repository containing ligands, receptors, cofactors, and their interactions with literature support in humans and mice. CellChat required gene expression data from cells as input and simulates the probability of cell‐cell communication by combining gene expression with prior knowledge of interactions between signaling ligands and receptors and their co‐factors. Permutation tests were used to identify significant interactions between two groups of cells and to calculate significant p‐values. We introduced the ligand and receptor database of mouse data in cellchatDB, and used Cellchat to analyze the cell communication between endothelial cells and smooth muscle cells at different stages to explore the possible ligand‐receptor communication signaling pathways and interactions between them. In the ligand‐receptor cell communication bubble diagram, the probability of ligand‐receptor interaction in each signaling pathway was plotted by the combination of p‐value and communication strength (Commun.Prob), which can visually show the difference of ligand‐receptor communication signaling pathways between endothelial cells and smooth muscle cells at different stages.

### Data Analysis of Ubiquitin Score

Seurat package in AddModuleScore function module was used to calculate the single cell in a particular gene set score. The ubiquitinated gene set was used as input, and used the AddModuleScore function to calculate the enrichment Score on the single‐cell data, where the score was the mean expression of the ubiquitinated gene set calculated in each cell. Finally, the mean enrichment score of each group was calculated according to different periods for visual display.

### The Measurement of EC Clusters by DotPlot

The DotPlot function was also used in the Seurat package to visually display the expression intensity and frequency of a specific gene between different groups or different clusters. The size of the dot in the plot indicated the percentage of the number of cells expressing the gene in a group, and the color of the dot indicated the average expression level of the gene in all cells in a group. We used TRIM35 drawn DotPlot function gene expression levels in different groups bubble chart to display the gene expression differences in different periods.

### Study Population

*UK Biobank*: UK Biobank, a prospective cohort study previously described in detail, recruited more than 500 000 adults living in the United Kingdom aged 40 to 69 years between 2006 and 2010. Briefly, the extensive baseline and follow‐up data was generated from questionnaires, health records, physical measurements, and biological samples. We included individuals with recorded CABG surgery at baseline (*N* = 4489) in UKB (Table S1, Supporting Information), and then excluded participants diagnosed with major adverse coronary event (MACE) at baseline (*N* = 1343), with missing information on plasma protein MMP10 levels (*N* = 2794). Finally, there were 352 participants included in our analyses. All participants provided informed written consent.

The outcomes of this study encompassed CVD mortality, and the incidence of MACE including MI, stroke, and heart failure. All residents in England, Scotland, and Wales were linked to electronic health records by using a unique National Health Service identification number. The subjects were followed from baseline until the date of first MACE diagnosis, date of death, or the last date of the follow‐up (October 31, 2022). The diagnosis for incident CVD and MACE was coded according to the WHO International Classification of Diseases Tenth Revision (ICD‐10).

Information on socioeconomic status (average household income) and lifestyle factors (smoking, alcohol consumption) was collected by self‐administered questionnaire. Demographic information on age, sex, body mass index (BMI), and systolic blood pressure (SBP) in participants was collected by trained nurses during the baseline assessment center visit. Diabetes was defined as current use of anti‐diabetic drugs, or self‐reported physician‐diagnosed diabetes (ICD‐10) or glucose ≥7.0mmol L^−1^. Moreover, 33 chronic comorbidities at baseline were defined according to ICD‐10.

Cox proportional hazards models were used to estimate the hazard ratios (HR) with a 95% confidence interval (CI) of incident MACE and mortality. The fully adjusted models were adjusted for age, sex, bmi, SBP, smoking, drinking, average household income, diabetes, and number of comorbidities. Linear regression and logistics regression analyses were performed to investigate whether MMP10 is associated with the risk factors for adverse outcomes in CABG patients with the standardized coefficients (β) and 95% confidence interval (CI). We evaluated the predictability of MMP10 on 10‐year MACE risk and CVD mortality by receiver operating characteristic (ROC) curves and their respective areas under the curve (AUC).

*Framingham Offspring Study*: The Framingham Offspring Study (FHS) enrolled 5214 offspring of the original FHS cohort and their spouses in 1971. Participants of the FHS Offspring cohort underwent standardized examinations and completed questionnaires with a study physician every 4 to 8 years. Beyond the clinical characteristics gathered during these examinations, the FHS data encompassed measurements of 88 serum protein immunoassays, taken at the seventh examination as part of the Systems Approach to Biomarker Research in Cardiovascular Disease Initiative, a sub‐study of the FHS. The Framingham Offspring Study was approved by the Boston University Medical Center Institutional Review Board (IRB).

The thoracic aortic calcium scores (TAC) were read by Massachusetts General Hospital Cardiovascular CT (CVCT) Core Lab on TeraRecon and saved as an Access database. It was electronically sent to Framingham Heart Study. The TAC score is considered as the most common form of extra‐coronary calcification. Our analysis utilized data from examination 7 (1998‐2001) of participants with a valid MMP10 measurement (*N* = 3,947), excluding those without a TAC measurement at follow‐up (*N* = 3,732). Ultimately, our analyses included 126 participants (Table S2, Supporting Information).

Multivariate analyses of MMP10 levels, sex, BMI, current smoking status, and blood pressure levels were conducted using logistic regression. Model 1 incorporated MMP10 levels; model 2 included MMP10 levels, sex, and BMI; and model 3 included MMP10 levels, sex, BMI, current smoking status, and blood pressure levels. For the analysis, we examined the change in MMP10 levels per unit of TAC per 1/1000 units. All participants provided informed consent prior to data collection.

### Statistical Analyses

All results presented as follows: mean ± standard error (SEM) for continuous variables, median (25th – 75th quartiles) for continuous variables, and n (%) for categorical variables. For comparisons between two groups, significance was assessed using unpaired two‐tailed Student's *t*‐test and unpaired *t*‐test with Welch's Correction. For comparisons among multiple groups, analysis of variance (ANOVA) was performed using R (version 4.1.1, Vienna, Austria). *p* < 0.05 was considered statistically significant. Statistical analyses were performed using R (version 4.1.1) and Stata (version MP 17.0). All experiments were conducted in three separate replications independently.

## Conflict of Interest

The authors declare no conflict of interest.

## Supporting information



Supporting Information

## Data Availability

The data that support the findings of this study are openly available in [Impact of Local Alloimmunity and Recipient Cells in Transplant Arteriosclerosis] at [https://doi.org/10.1161/CIRCRESAHA.119.316470], reference number [6].
