# Supplementary material for: Endothelial TRIM35‐Regulated MMP10 Release Exacerbates Calcification of Vascular Grafts
Source: Adv Sci (Weinh). 2025 Jan 27;12(11):2409641. doi: 10.1002/advs.202409641 (PMC11923891; doi:10.1002/advs.202409641)
Supplement: Supplementary file 1 — Supporting Information [file ADVS-12-2409641-s001.pdf]

ADVANCED SCIENCE
Open Access

Supporting Information

for *Adv. Sci.*, DOI 10.1002/advs.202409641

Endothelial TRIM35-Regulated MMP10 Release Exacerbates Calcification of Vascular Grafts

Yiming Leng, Wei Wang, Jun Lu, Jingyuan Chen, Xuliang Chen, Yalan Li, Jie Wang, Yuanyuan Liu, Qian Tan, Wenjing Yang, Youxiang Jiang, Peiyuan Huang, Jingjing Cai, Hong Yuan, Liang Weng, Qingbo Xu and Yao Lu**

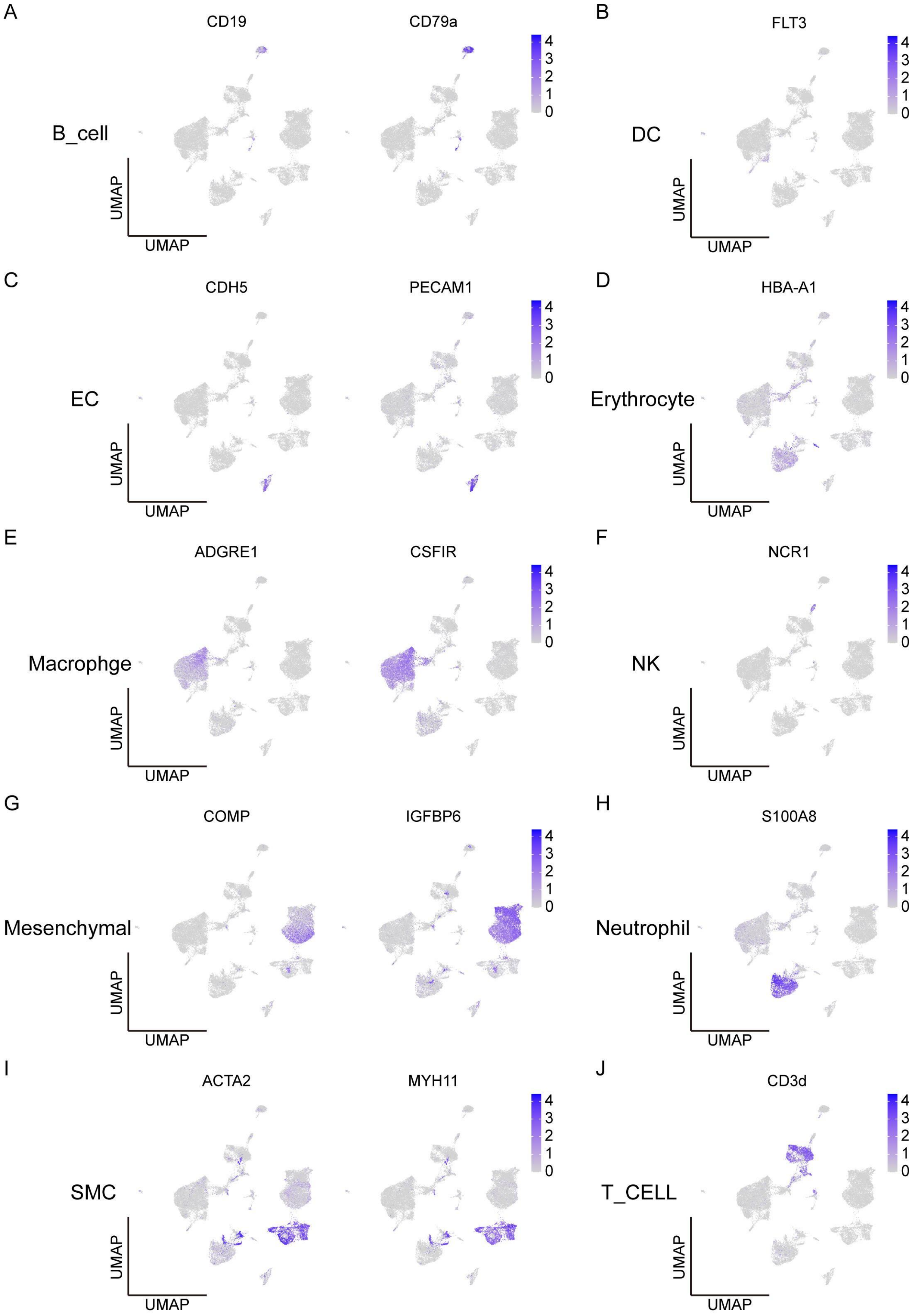

Figure S1. Graft Cell Clusters' Signature Gene Expression Profile.
Marker gene expression signature of B cell (A), DC (B), EC (C), Erythrocyte (D), Macrophage (E), NK (F), Mesenchymal (G), Neutrophil (H), SMC (I) and T cell (J).

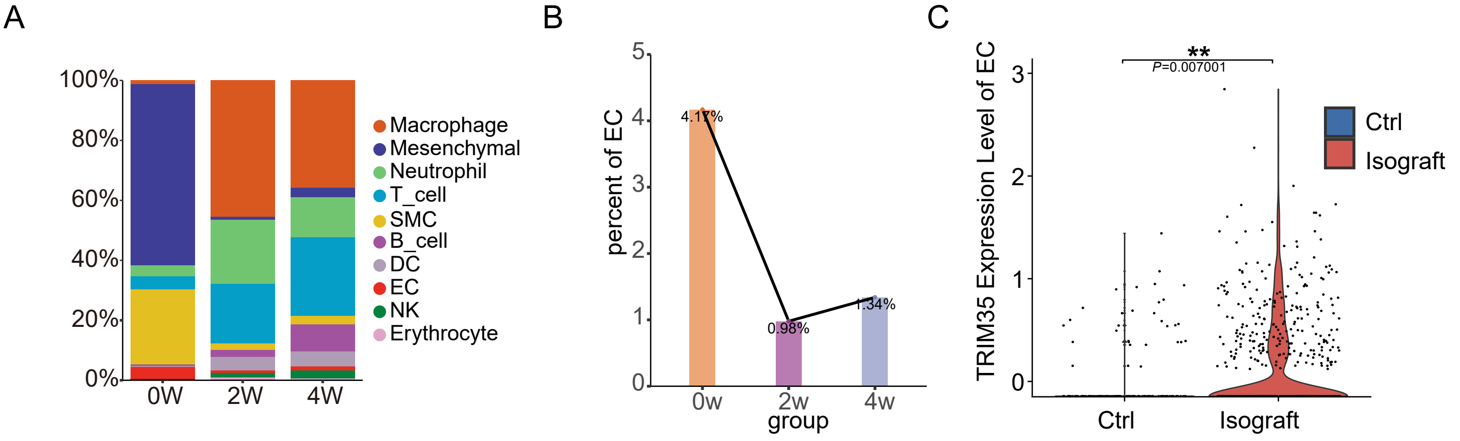

Figure S2. **Proportions of major cell types and dynamic changes of EC in Single-cell RNA-seq** A: Bar chart displaying the proportion of major cell types among different groups. B: Bar chart showing the proportion of EC cluster in 0W, 2W and 4W group.C: The violin plots of TRIM35 expression in vascular EC cluster. Data are means and SEM, *P < 0.05, **P < 0.01, ***P < 0.001, ****P < 0.0001.

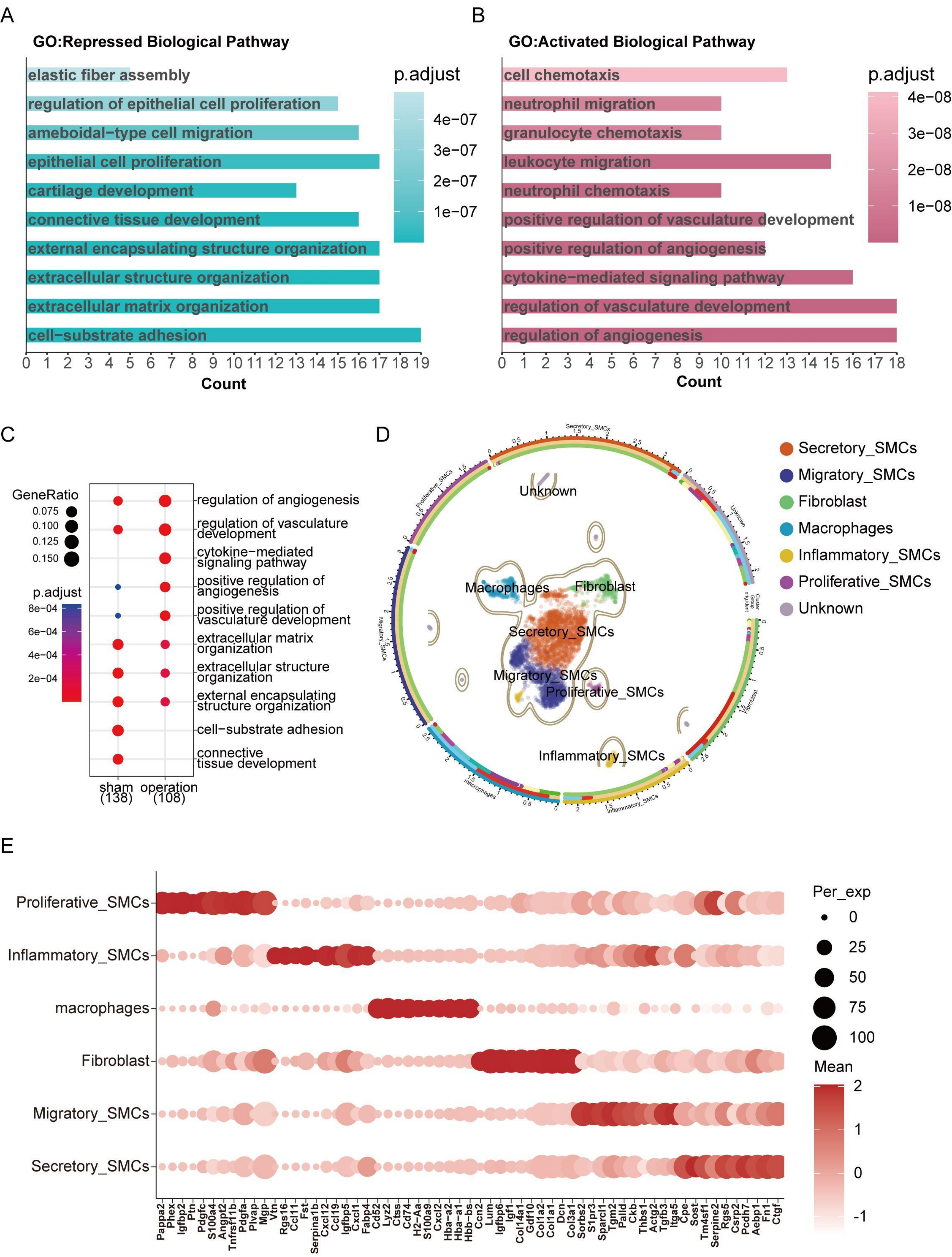

Figure S3. Changes of Endothelial Function and SMC Phenotypic Switching Spectrum. A and B. Gene set enrichment analyses displaying top 10 enriched downregulated (A) or upregulated (B) genes ontology biological processes (GOBP) in EC cluster. C. GOBP analysis displaying top 10 enriched pathways between sham group and operation group. D. UMAP plot for 7 subclusters of SMC in the aortic graft, n=4,613 cells. E. Dot plots showing expression levels of selected indicated subclusters.

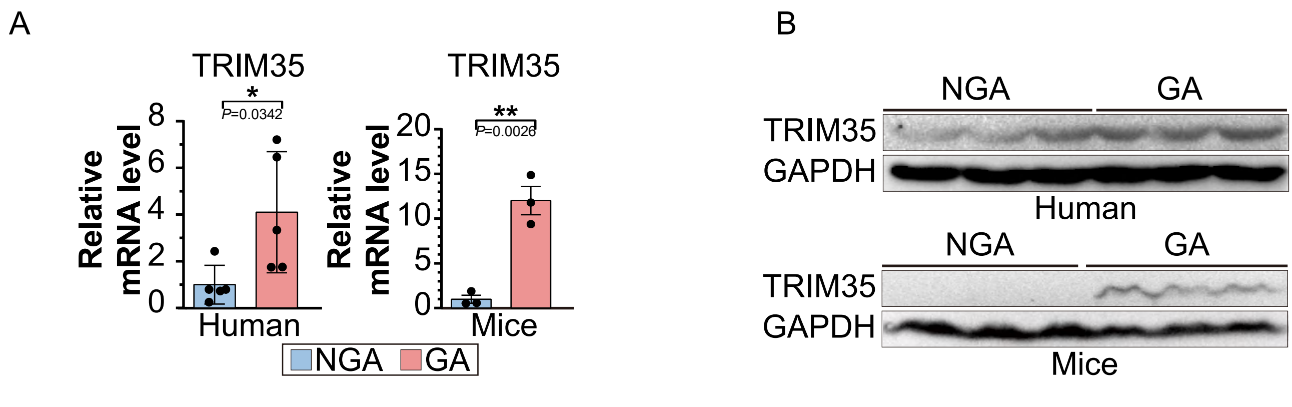

Figure S4. Upregulated transcription and translation of TRIM35 in EC of GA grafts.

A. Quantitative Polymerase Chain Reaction (qPCR) illustrating TRIM35 RNA expression in arteries from human and mice with GA or NGA, n=5 in human group and n=3 in mice group. B. Western-blotting illustrating TRIM35 protein expression in arteries from human and mice with GA or NGA, n=3 in each group. Data are means and SEM; *P<0.05, **P<0.01, ***P<0.001.

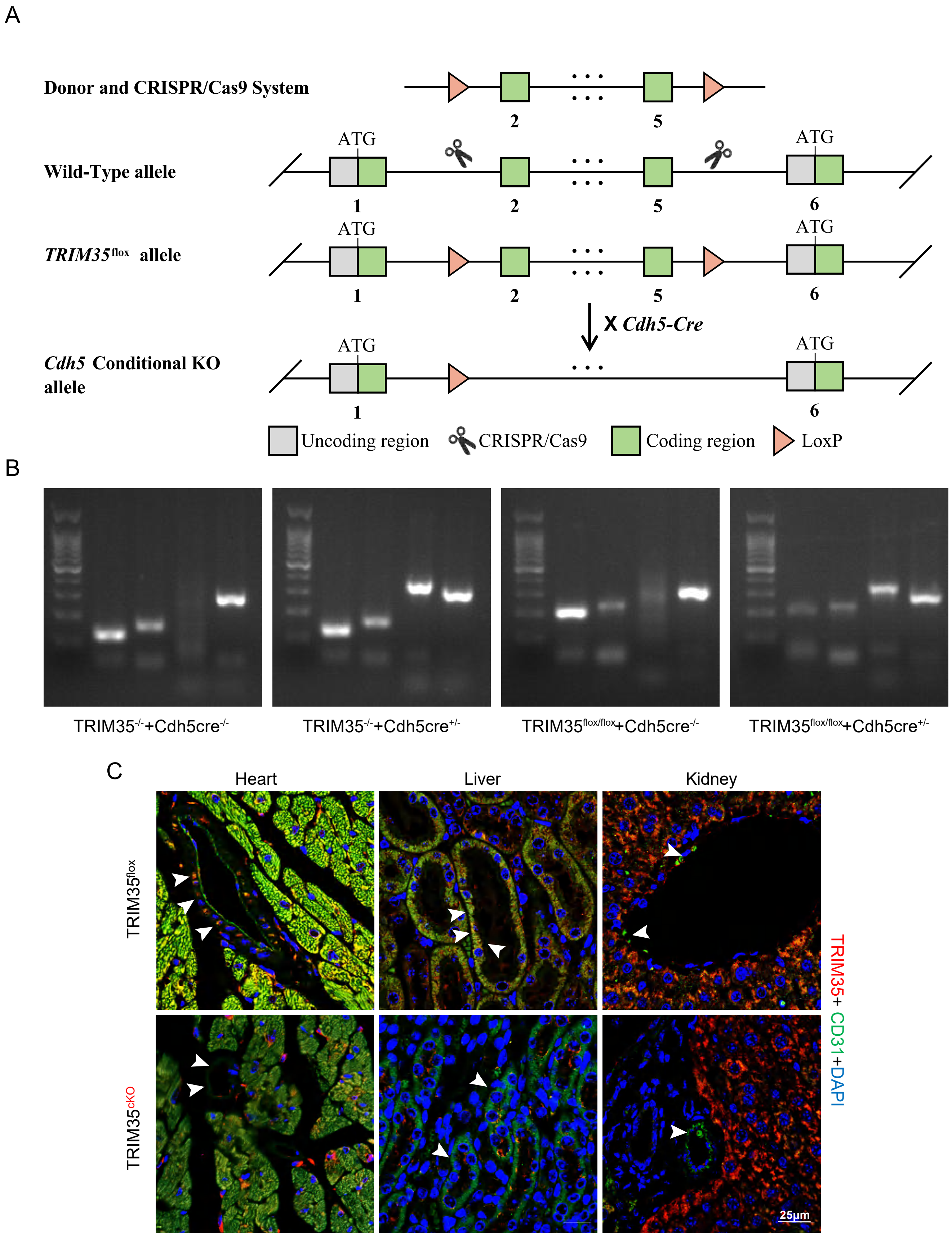

Figure S5. Strategy and Validation of Endothelial-Specific Deletion of TRIM35.
A. Schematic diagram of the TRIM35cKO mice construction. B. Genotyping of wild-type mice, Cdh5cre mice, TRIM35flox mice and TRIM35cKO mice. C. Heart, liver and kidney from TRIM35flox or TRIM35cKO mice were stained by anti-TRIM35 (green) and anti-CD31 (red) antibodies to validate the knockout efficiency.

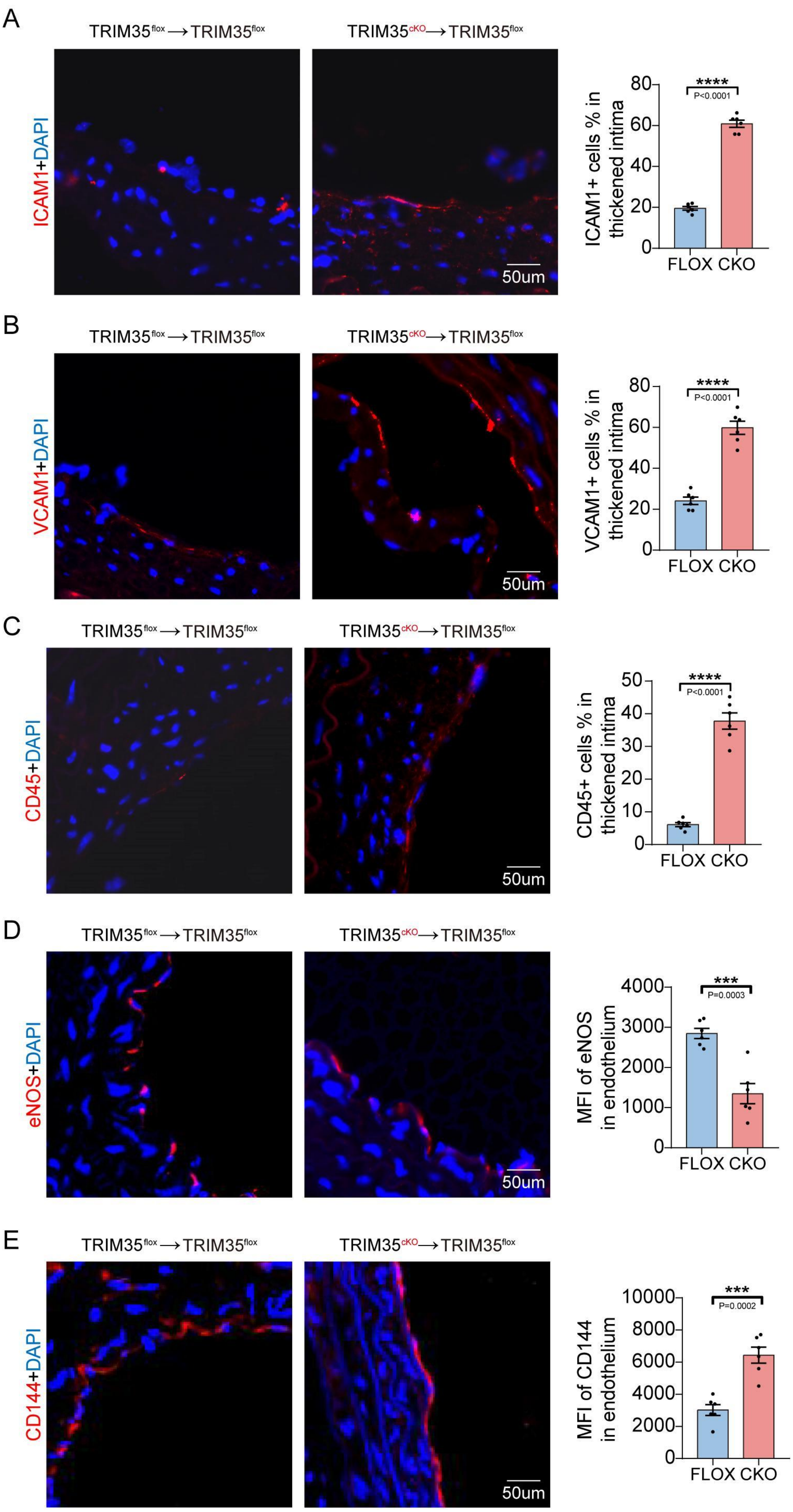

Figure S6. Endothelial-Specific Deletion of TRIM35 Increased Graft Inflammation and Slowed Endothelial Recovery.

A-C. IF staining for ICAM1 (A), VCAM1 (B) and CD45 (C) of graft arteries from TRIM35flox or TRIM35cKO mice, n=6 in each group. D and E. IF staining for eNOS (D) and CD144 (E) of graft arteries from TRIM35flox or TRIM35cKO mice, n=6 in each group. Data are means and SEM, ***P < 0.001, ****P < 0.0001. MFI, mean fluorescence intensity.

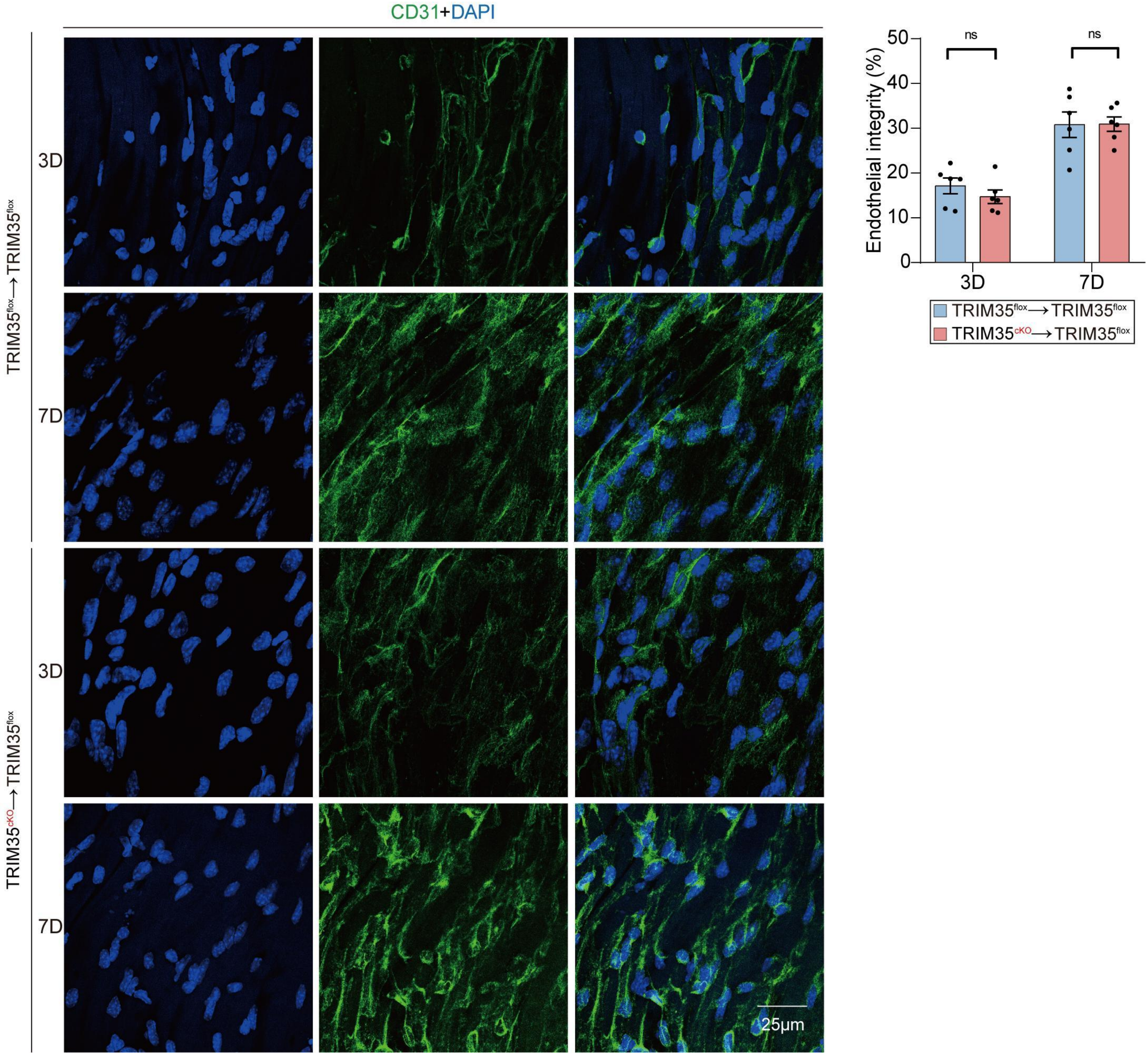

Figure S7. Deletion of TRIM35 in EC Did not Aggravate Endothelial Permeability.
En face staining for CD31 of graft arteries from TRIM35flox or TRIM35cKO mice 3 or 7 days after surgery. N=6 in each group. Data are means and SEM.

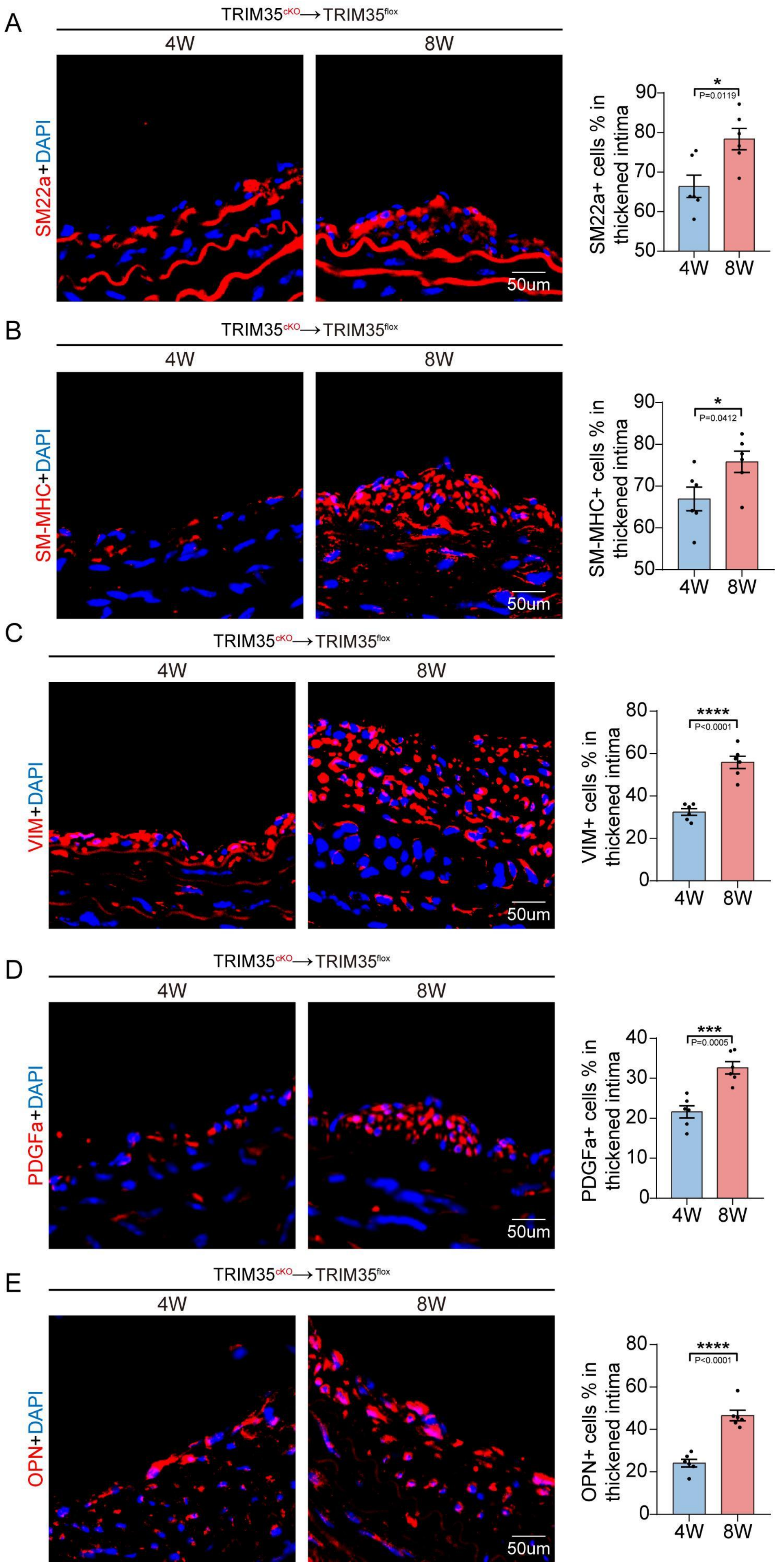

Figure S8. Deletion of TRIM35 in EC Promoted SMC Phenotypic Switching.
A-E. IF staining for SM22a (A), SM-MHC (B), VIMENTIN (C), PDGFa (D) and OPN (E) of graft arteries from TRIM35cKO mice, n=6 in each group. Data are means and SEM, *P < 0.05, ***P < 0.001, ****P < 0.0001.

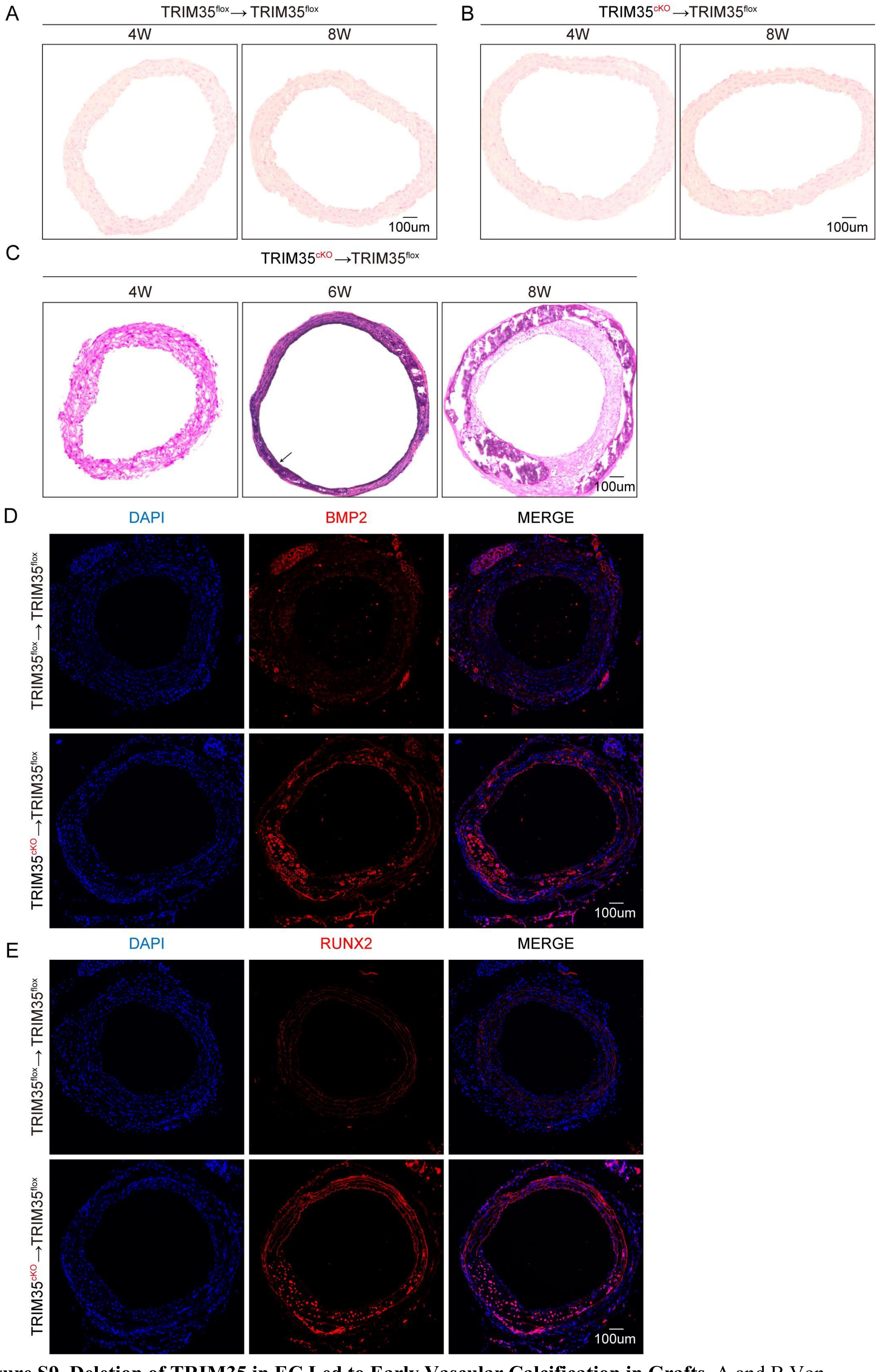

Figure S9. Deletion of TRIM35 in EC Led to Early Vascular Calcification in Grafts. A and B.Von Kossa and Nuclear Fast Red staining for unthickened area in graft arteries from TRIM35flox (A) or TRIM35cKO (B) mice. C. HE staining of isograft arteries from TRIM35cKO mice 4W, 6W and 8W after surgery. D and E. IF staining for BMP2 (D) and RUNX2 (E) of graft arteries from TRIM35flox or TRIM35cKO mice 8W after surgery.

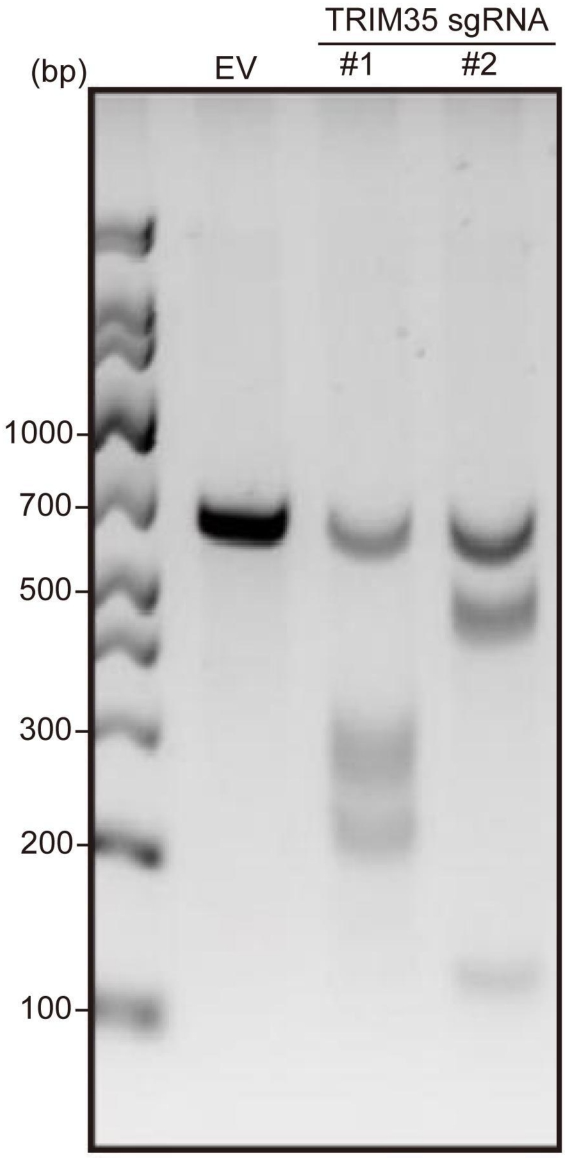

Figure S10. TRIM35 Knockout Validation in HUVEC Cell Line.
Restriction enzyme digestion and agarose gel electrophoresis was performed to detect TRIM35 KO HUVEC cell lines construction.

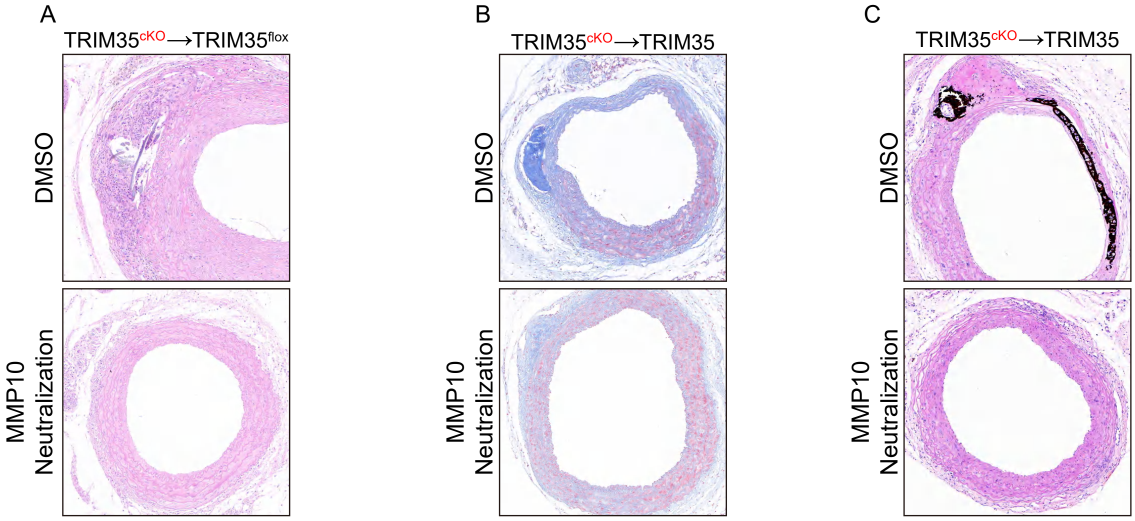

Figure S11. Neutralizing MMP10 alleviates vascular calcification.
HE staining (A), Masson staining (B), and Von Kossa (C) of isograft arteries from 8W TRIM35cKO mice locally administered MMP10 neutralizing antibody or DMSO.

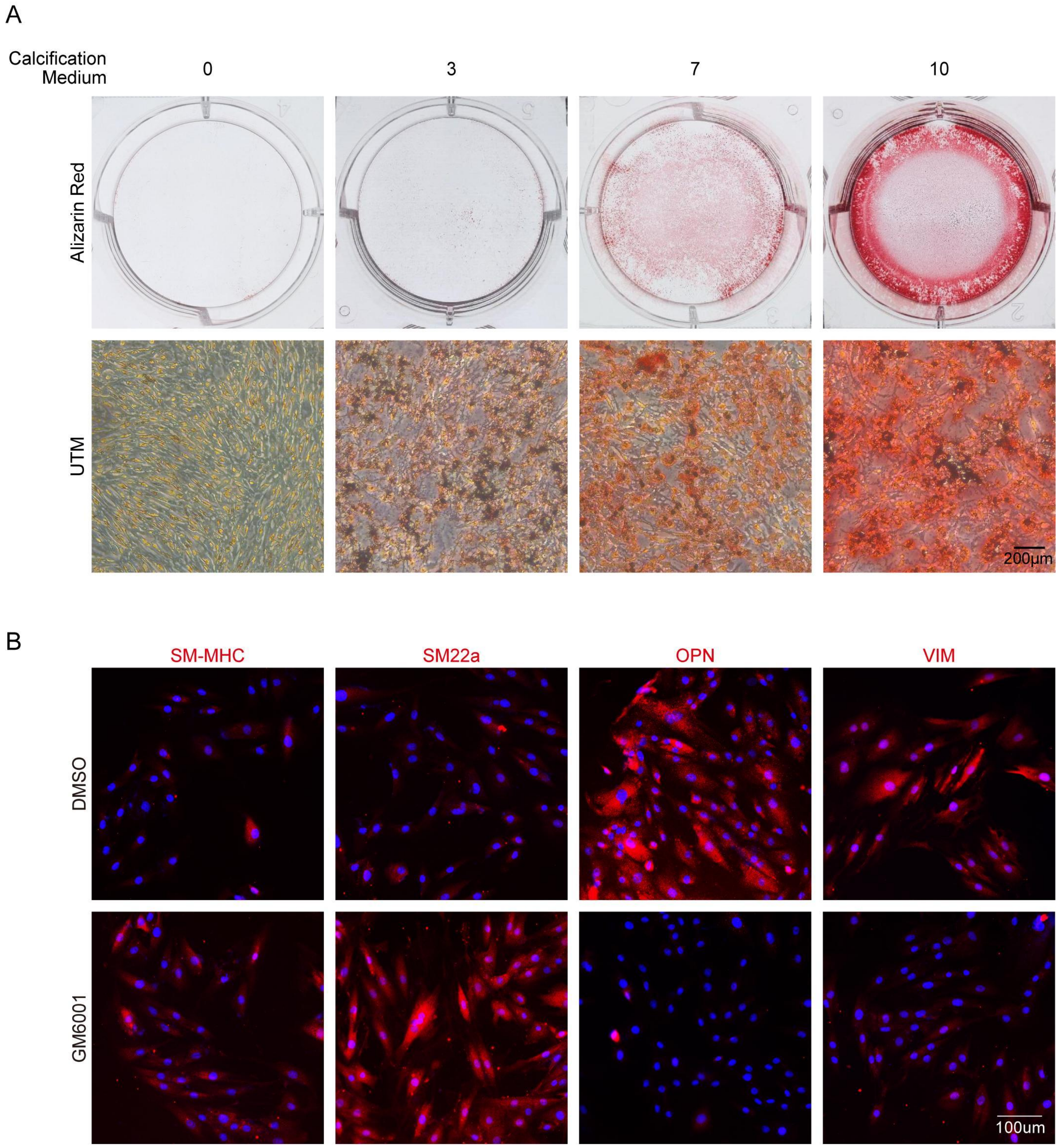

Figure S12. **Inhibition of MMP10 Activity Alleviated SMC Phenotypic Switching.** A. Alizarin Red staining for calcium nodules in primary VSMC stimulated by Calcification medium for 0d, 3d, 7d and 10d. B. Immunocytochemistry (ICC) was conducted in primary VSMC treated with conditioned medium generated from TRIM35cKO pEC and DMSO or GM6001 and followed by detection with anti-SM-MHC, anti- SM22a, anti-OPN and anti-VIMENTIN antibodies.

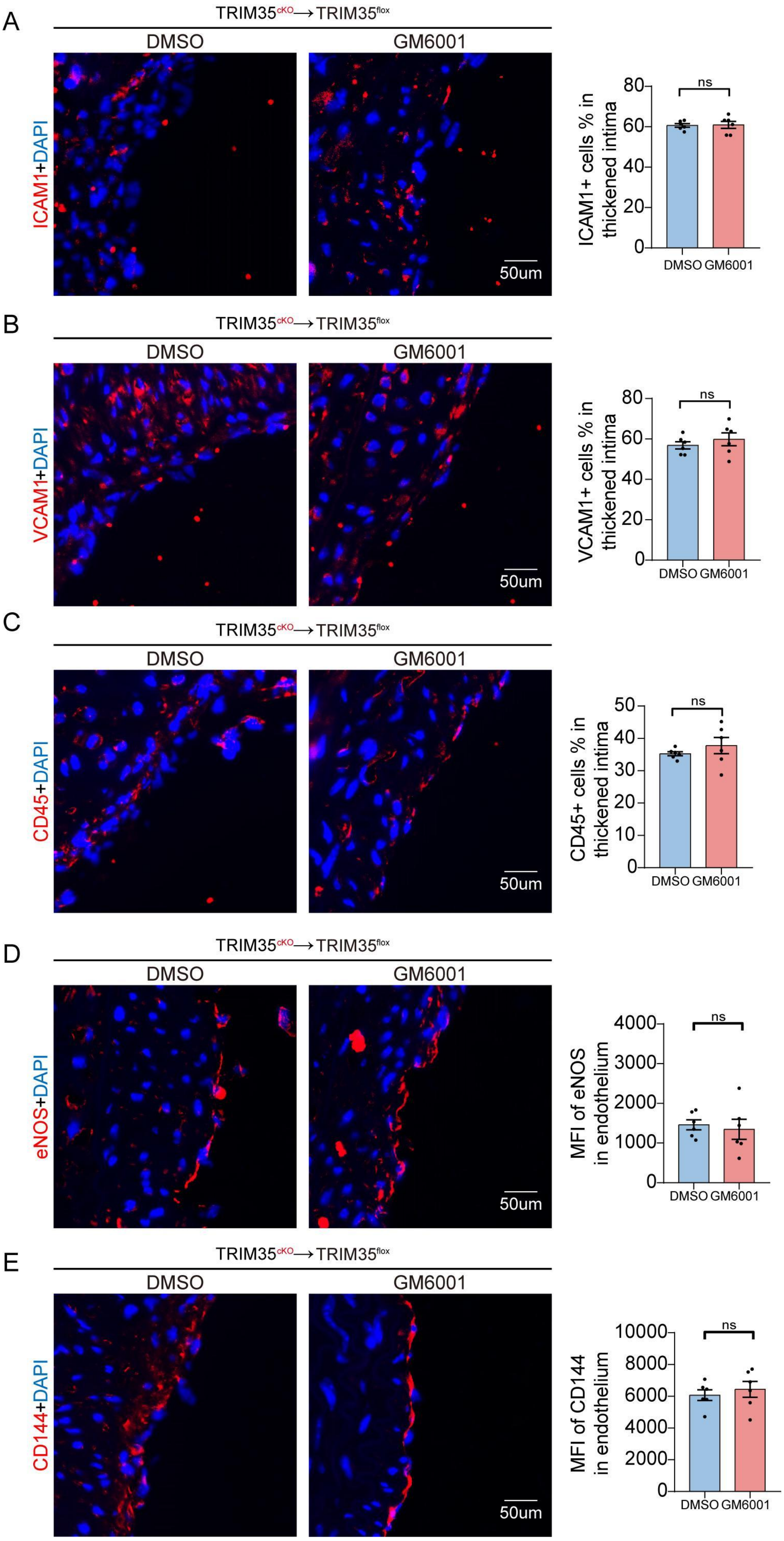

Figure S13. Inhibition of MMP10 Activity Attenuates Vascular Inflammation Caused by Endothelial TRIM35 Knockout.

A-C. IF staining for ICAM1 (A), VCAM1 (B) and CD45 (C) of graft arteries from TRIM35cKO mice locally administrated with DMSO or GM6001 for 8 weeks, n=6 in each group. C and D. IF staining for eNOS (C) and CD144 (D) of graft arteries from TRIM35cKO mice locally administrated with DMSO or GM6001 for 8 weeks, n=6 in each group. Data are means and SEM.

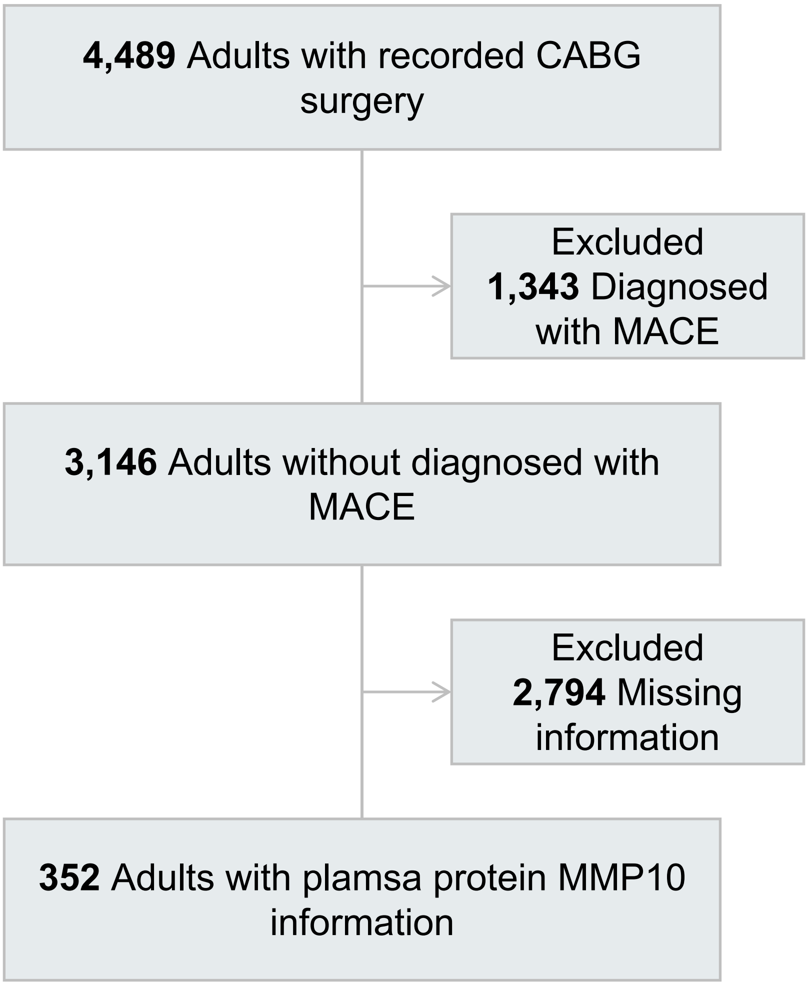

Figure S14. Workflow of participants in UK Biobank

CABG, Coronary artery bypass grafting; MACE, Major Adverse Cardiovascular Events.

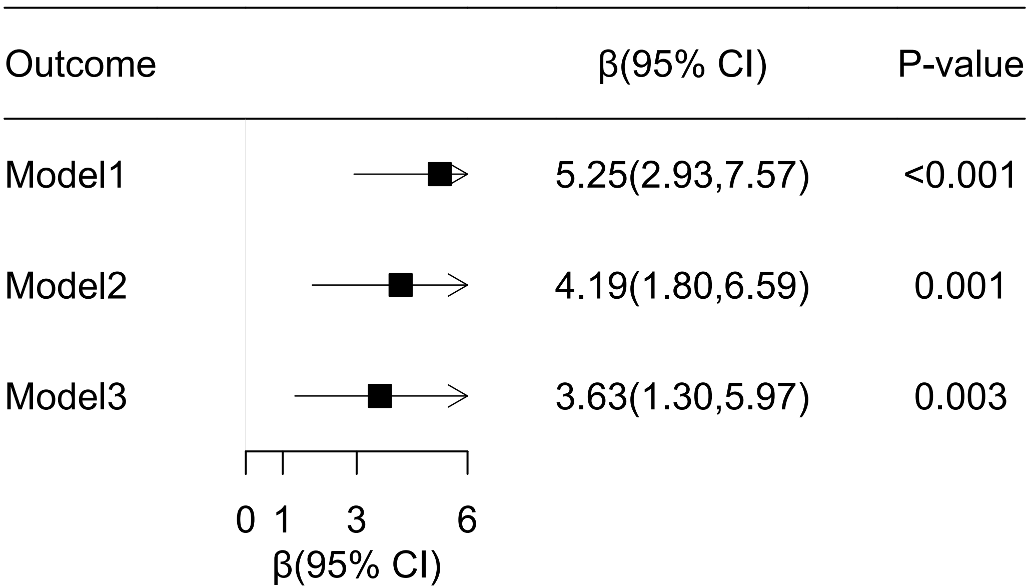

Figure S15. Framingham Offspring Study

Association between risk of TAC score and MMP10 in model1, 2 and 3.

TAC:The thoracic aortic calcium scores.

Model 1 incorporated MMP10 levels.

Model 2 included MMP10 levels, sex, and BMI.

Model 3 included MMP10 levels, sex, BMI, current smoking status, and blood pressure levels.

	Age	Gender (Male/Female)	Current smoker (True/False)	Former smoker (True/False)	SBP(mmHg)	DBP(mmHg)	Heart rate (beats per minute)	Tissue Source	Hypertension (True/False)
Patient 1	41	M	T	T	102	72	112	Anterior descending branch + LIMA	F
Patient 2	49	M	T	T	162	78	80	Left main branch+LIMA	T
Patient 3	74	M	T	T	120	68	51	Recurrent branch+LIMA	F
Patient 4	63	M	T	T	110	84	68	LIMA	F
Patient 5	71	M	F	T	118	67	70	Left main coronary artery	T
Patient 6	60	M	F	F	142	72	78	LIMA	T
Patient 7	38	F	F	F	165	63	94	Recurrent branch	T

Figure S16. Basic Information of Tissue Providers. Patient information and tissue source for CABG.
LIMA: left internal mammary artery

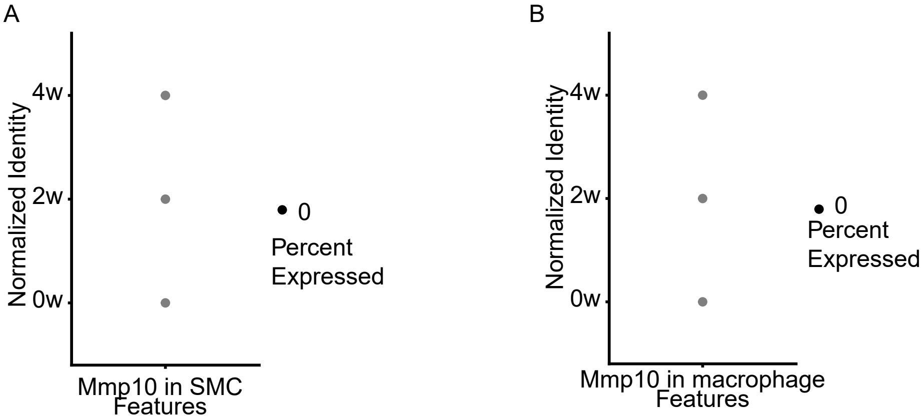

Figure S17. Single cell-seq analysis of MMP10 expression in SMC (A) and macrophages (B).

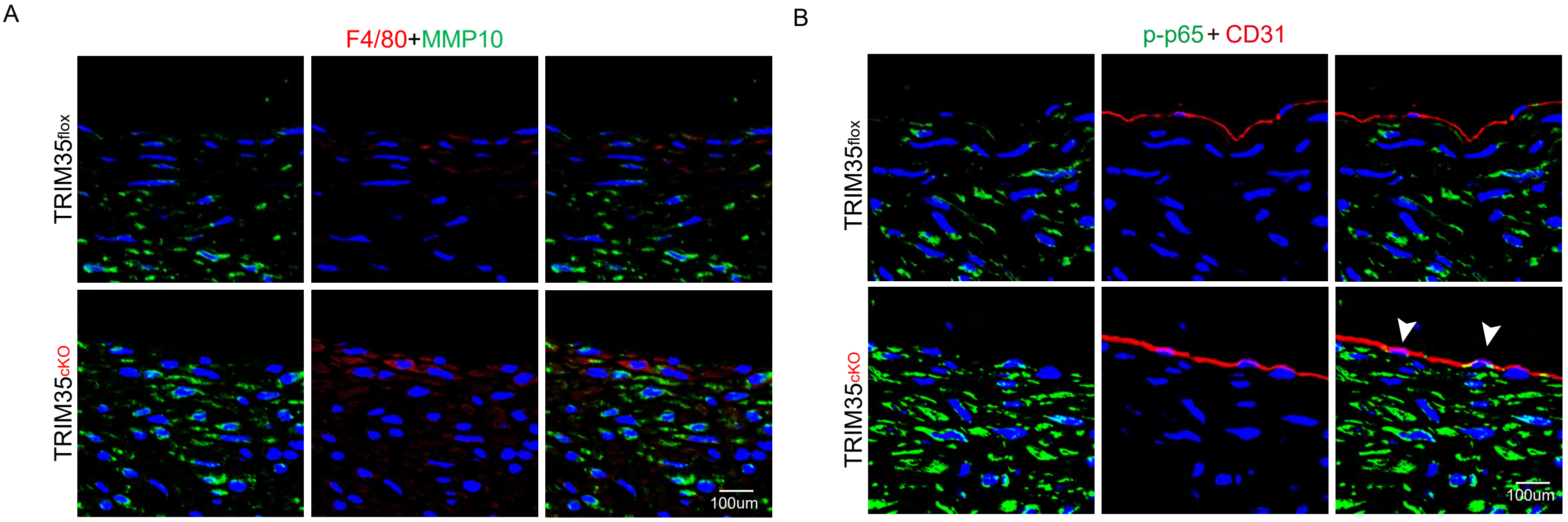

Figure S18. Validation of MMP10 in macrophage and canonical NF-kB pathway response.

A. IF staining for F4/80 (red) and MMP10 (green) of graft arteries from TRIM35flox or TRIM35cKO mice. B. IF staining for CD31 (red) and p65 phosphorylation (green) of graft arteries from TRIM35flox or TRIM35cKO mice.

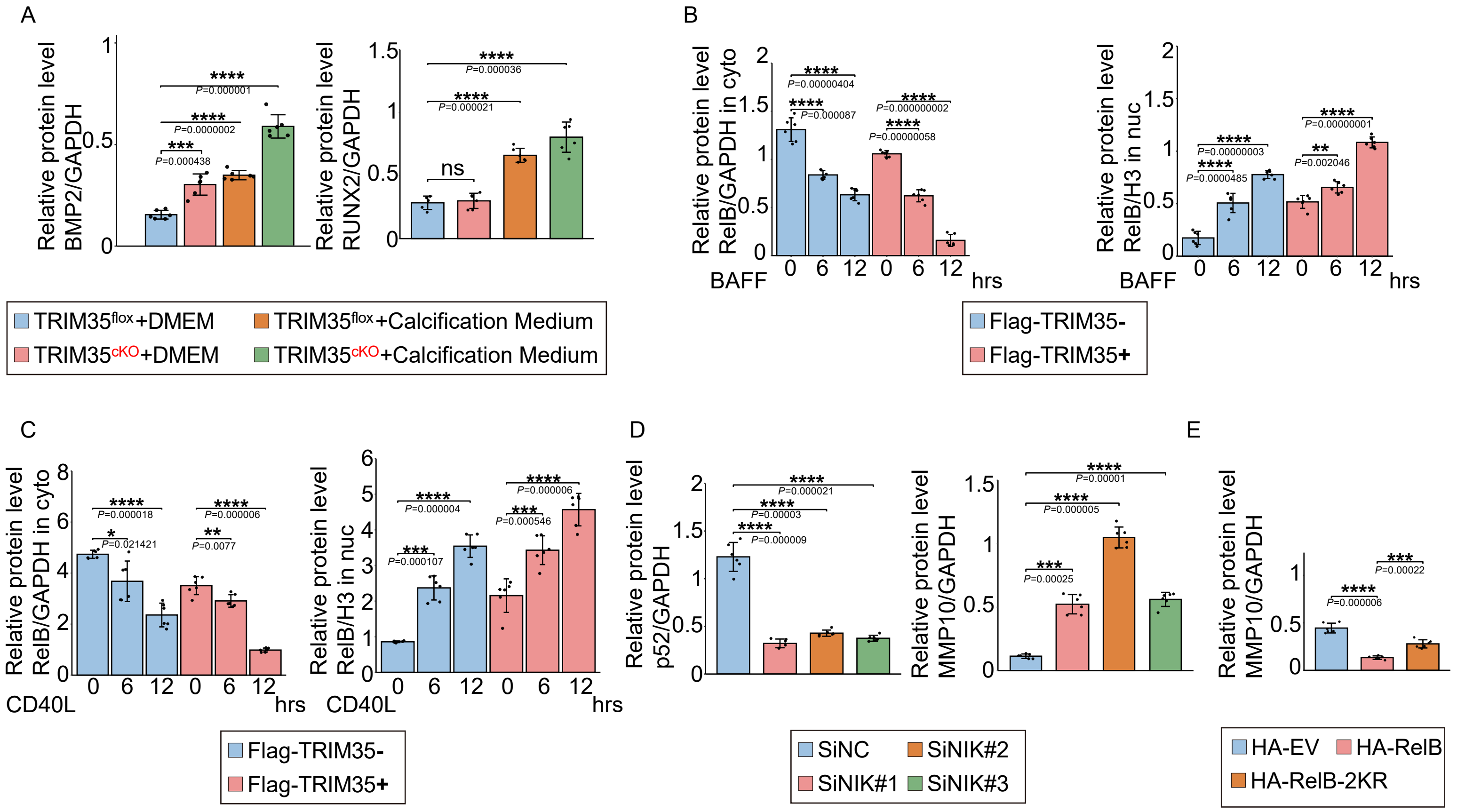

Figure S19. Quantification of western blot analyses for Figure 3H, Figure 6C, Figure 6D, Figure 6H and Figure 6I

A.Relative protein level of BMP2 and RUNX2 of Figure 3H. B. Relative protein level of RelB of Figure 6C. C. Relative protein level of RelB of Figure 6D. D. Relative protein level of p52 and MMP10 of Figure 6H. E. Relative protein level of MMP10 of Figure 6I.

Table S1 Baseline Characteristics of the UK biobank

Characteristics	Overall<br>352
Age (years)	66(62-68)
Male	298 (84.6%)
CVD mortality	52 (14.8%)
MACE	151 (42.9%)
BMI	29.1 (26.3-32.3)
SBP	139.8(129.2-152)
Smoking	
Never	104(29.6)
Previous	219(62.2)
Current	29(8.2)
Drinking	
Never	17(4.8)
Previous	13(3.7)
Current	322(91.5)
Average household income	
level 1, <£18 000	122(42.8)
level 2, £18 000–£30 999	92(32.3)
level 3, £31 000–£51 999	50(17.5)
level 4, £52 000–£100 000	15(5.3)
level 5, >£100 000	6(2.11)
Diabetes	98 (27.8%)
Number of comorbidities	3(2-5)

MACE, major adverse coronary event, including MI, stroke, and heart failure; BMI,body mass index; SBP,systolic blood pressure

Table S2 Baseline Characteristics of the Framingham Offspring Study

Characteristics	Overall<br>126
Age (years)	67(62-72)
Male	58 (46.0%)
BMI	29.2 (26.4-32.6)
Current smoking status	
YES	4 (3.1%)
NO	122 (96.9%)
DBP	71(61-78)
SBP	129 (120-143)

BMI,body mass index; DBP,diastolic blood pressure; SBP,systolic blood pressure

**REAGENTS**	**SOURCE**	**CATLOG**
**Antibodies**		
Rabbit Anti-TRIM35 antibody	Bioss	bs-9149R
Anti-TRIM35 antibody produced in rabbit	Sigma	HPA019647
Rabbit Anti-TRIM35/AF488 Conjugated antibody	Bioss	bs-9149R-AF488
Vimentin Rabbit pAb	Abclonal	A11952
Vimentin Monoclonal antibody	Proteintech	60330-1-Ig
Alexa Fluor® 488 Anti-Vimentin antibody [EPR3776]	Abcam	ab185030
Rabbit monoclonal [EPR21139-316] to Osteopontin	Abcam	ab214050
MMP10 Rabbit pAb	ABclonal	A3033
BMP2 Rabbit pAb	ABclonal	A0231
BMP2 Rabbit pAb	ABclonal	A12781
RUNX2 Rabbit mAb	ABclonal	A11753
Phospho-NF-kB p65/RelA-S536 mAb	ABclonal	AP0475
NF-kB p65/RelA Rabbit mAb	ABclonal	A19653
PDGFA Rabbit pAb	Abclonal	A17434
Transgelin (TAGLN) Rabbit mAb	Abclonal	A21209
Mouse anti HA-Tag mAb	ABclonal	AE008
SMMHC/MYH11 Rabbit mAb	ABclonal	A4064
purified Rat-anti-mouse CD31 Antibody	Biolegend	102502
purified Rat-anti-mouse CD102 Antibody	Biolegend	105602
Anti-Mouse CD31	Proteintech	65058-1-Ig
Alexa Fluor® 594 anti-mouse CD31 Antibody	Biolegend	102520
Rabbit Anti-MAP3K14/NFkB Inducing Kinase antibody	Bioss	bs-0074R
Biotin-SP-conjugated Affinipure Goat Anti-Rat IgG(H+L)	proteintech	SA00004-8
NFKB2 Polyclonal antibody	Proteintech	10409-2-AP
transgelin/SM22 Polyclonal antibody	Proteintech	10493-1-AP
RELB Polyclonal antibody	Proteintech	25027-1-AP
F4/80 antibody	Abcam	ab16911
MMP10 antibody (for neutralizing)	R&D	MAB9101
GAPDH Monoclonal antibody	Proteintech	60004-1-Ig
FLAG antibody	Proteintech	20543-1-AP
GST antibody	Proteintech	66001-2-Ig
anti-Mouse HRP	Proteintech	SA00001-1
anti-Rabbit HRP	Proteintech	SA00001-2
Donkey anti-Rabbit Secondary Antibody, Alexa Fluor™ 488	Invitrogen	A-21206
Donkey anti-Rat Secondary Antibody, Alexa Fluor™ 594	Invitrogen	A-21209
Donkey anti-Rabbit Secondary Antibody, Alexa Fluor™ 594	Invitrogen	A-21207

Chemicals, Recombinant proteins and Kits		
DMEM	Gibco	11995065
ECM	Sciencell	1001
FBS	Gibco	10100147
Endothelial cell medium	sciencell	1001
0.25% Trypsin-EDTA	Gibco	25200072
Penicillin / Streptomycin / Amphotericin B,sterile solution	Basal Media	S120JV
CryoStor® CS10	Biolife Solutions	07959
Puromycin	Thermo Fisher Scientific	A1113803
Bovine serum albumin, fraction V, heat shock isolation	Sangon Biotech	A600332
Primary Antibody Dilution Buffer	Beyotime	P0023A
Can Get SignalTM Immunoreaction Enhancer Solution 1	TOYOBO	NKB-201
Tissue digestive enzyme II	Precedo	PRS-TDE-II
Streptavidin magnetic beads	Proteintech	MS001
Immobilon Western HRP Substrate Kit	Millipore	WBKLS0500
Protease Inhibitor Cocktail Tablets	Roche	11836170001
PhosSTOP™	Roche	4906837001
PierceTM BCA Protein Assay Kit	Thermo Fisher Scientific	23225
PVDF membrane	Millipore	IPVH00010
Lipofectamine 3000	Invitrogen	L3000008
LipoRNAi™ Transfection Reagent	Beyotime	C0535
Water-DEPC Treated Water	Sangon Biotech	B501005
iTaq™ Universal SYBR® Green Supermix	BIO-RAD	1725125
RT Reverse Transcription Kit	Thermo Fisher Scientific	K1691
PrimeSTAR® GXL DNA Polymerase	Takara	R050A
Phenol, saturated with water	Sangon Biotech	A504195
Pluronic F-127	Sigma	P2443
DAPI	Servicebio	G1012
O.C.T.	Sakura	4583
FluoroshieldTM mounting medium	Sigma	F6182
Triton X-100	Sigma	T8787
Recombinant Stromelysin-2(MMP10)	CUSABIO	CSB-YP014657HU
Sterile calcium chloride solution (1mol/L)	Solarbio	G0070

β-Glycerol phosphate disodium salt pentahydrate	Solarbio	G8100
Alizarin Red S Solution, 1%, pH4.2	Solarbio	G1452
Alizarin Red S Solution, 0.2%	Abiowell	AWI0292
Evan's Blue Stain Solution, 0.5%	Solarbio	314-13-6
Alkaline Phosphatase Assay Kit	Beyotime	P0321S
Human MMP-10(Matrix Metalloproteinase 10) ELISA Kit	MULTISCIENCES	XY9H3367
Masson's Trichrome Stain Kit	Abiowell	AWI0253a
Calcium Stain Kit (Von Kossa Method)	Solarbio	G3282
Picrosirius Red Staining Kit	Solarbio	AWI0626
GM6001	Absin	142880-36-2
**Software**		
Image J	https://imagej.nih.gov/ij/	
Graphpad Prism 8 (GraphPad)	https://www.graphpad-prism.cn/	
Image Lab	https://info.bio-rad.com/ww-image-lab-lp.html	
R Studio	https://posit.co/download/rstudio-desktop/	

PCR No.	Primer No.	Sequence	Band Size	Gene
①5’arm	T004712-F1-Cdh5	GGGCAGTCTGGTACTTCCAAGCT	WT:0bp	Cdh5
	T004712-R1-Cdh5	CCTCGTGTGTACTGGTCAAGTGCTC	Targeted:318bp	
②WT	T004712-F2-Cdh5	GGGCAGTCTGGTACTTCCAAGCT	WT:285bp	
	T004712-R2-Cdh5	ATATCCCCTTGTTCCCTTTCTGC	Targeted:0bp	
①5’arm+ WT	JS00275-Trim35-5wt-tF1	GCCAGACAATCAGTGAGTTGG	WT:122bp	TRIM35
	JS00275-Trim35-5wt-tR1	CTGGAATGTCCACTCACGCTG	Fl:179bp	
②3’arm+WT	JS00275-Trim35-3wt-tF1	CAACTCTCTCCTGCCAGTGTG	WT:154bp	
	JS00275-Trim35-3wt-tR1	TCTGTTACAGCAGCCAAGGAGAC	FL:211bp	

Abbreviation:

CVD	cardiovascular disease
CABG	coronary artery bypass graft surgery
EC	endothelial cells
VSMC	vascular smooth muscle cells
TRIM35	tripartite motif 35
ECM	extracellular matrix
HUVEC	human umbilical vein endothelial cells
OPN	osteopontin
scRNA-seq	single-cell RNA sequencing
eNOS	endothelial nitric oxide synthase
CDH5	cadherin-5
ICAM1	intercellular adhesion molecule 1
VCAM1	vascular cell adhesion protein 1
MMP10	matrix metalloproteinase 10
BMP2	bone morphogenetic protein 2
RUNX2	Runt-related transcription factor 2
NF-κB	nuclear factor kappa-B
RelB	transcription factor RelB
UB	ubiquitin
BAFF	B-cell-activating factor receptor
CD40L	CD40 ligand
MACE	major adverse cardiovascular events
